# Sesquiterpenoids from *Dysoxylum acutangulum* Miq. and *Dysoxylum cauliflorum* Hiern Twigs: Antibiofilm Activity Against *Streptococcus mutans*

**DOI:** 10.3390/molecules31111893

**Published:** 2026-06-01

**Authors:** Risyandi Anwar, Hikma Ainazzahra, Citra Nisa Ul Inayah, Al Arofatus Naini, Endang Juliansyah, Elpri Eka Permadi, Aditya Nugroho, Kindi Farabi, Unang Supratman

**Affiliations:** 1 Herbal Medicine Research, Department of Pediatric Dentistry, Faculty of Dental Medicine, University of Muhammadiyah Semarang, Semarang 50272, Indonesia; 2 Department of Chemistry, Faculty of Mathematics and Natural Sciences, Universitas Padjadjaran, Jl. Raya Bandung–Sumedang Km. 21, Jatinangor, Sumedang 45363, Indonesia; hikma21001@mail.unpad.ac.id (H.A.); citra21003@mail.unpad.ac.id (C.N.U.I.); endang18001@mail.unpad.ac.id (E.J.); kindi.farabi@unpad.ac.id (K.F.); unang.supratman@unpad.ac.id (U.S.); 3 Research Center for Pharmaceutical Ingredients and Traditional Medicine, National Research and Innovation Agency (BRIN), Cibinong Science Center Complex-BRIN, Cibinong, Bogor 16911, Indonesia; alar002@brin.go.id (A.A.N.); elpri.eka.permadi@brin.go.id (E.E.P.); 4 Research Center for Applied Botany, National Research and Innovation Agency (BRIN), Cibinong Science Center Complex-BRIN, Cibinong, Bogor 16911, Indonesia; adit035@brin.go.id; 5 Central Laboratory, Universitas Padjadjaran, Jl. Raya Bandung–Sumedang Km. 21, Jatinangor, Sumedang 45363, Indonesia

**Keywords:** sesquiterpenoids, *Dysoxylum*, antibiofilm, *Streptococcus mutans*

## Abstract

Sesquiterpenoids are widely distributed in the genus *Dysoxylum* (Meliaceae) and have attracted attention because of their diverse biological activities, including antibiofilm properties. This study aimed to isolate and characterize sesquiterpenoids from the *n*-hexane extracts of *Dysoxylum acutangulum* Miq. and *Dysoxylum cauliflorum* Hiern twigs, followed by evaluation of their antibiofilm activity against *Streptococcus mutans*. Chromatographic separation of the *n*-hexane extract of *D. acutangulum* afforded spathulenol (**1**), β-caryophyllene oxide (**3**), and a mixture of epimeric sesquiterpenoids consisting of 10-oxo-isodauc-3-en-15-al (**2a**, major) and sinulin A (**2b**, minor), while humulene diepoxide A (**4**) was isolated from *D. cauliflorum*. Structural elucidation was carried out using MS, IR, and extensive NMR spectroscopic analyses, and by comparison with reported data. The major and minor epimers of compound **2** were distinguished based on diagnostic NMR signal intensities and comparison with literature data. Notably, all isolated compounds are reported herein for the first time from their respective *Dysoxylum* species. Compound **1**, the epimeric mixture **2a**/**2b**, and compound **4** exhibited weak antibiofilm activity against *S. mutans*, with minimum biofilm inhibitory concentration (MBIC) values ranging from 250 to 500 µg/mL, whereas chlorhexidine exhibited superior antibiofilm activity at 62.5 µg/mL. Molecular docking against Sortase A and GtfB revealed moderate binding affinities and suggested plausible interactions with biofilm-associated targets. These findings provide additional chemotaxonomic information on *Dysoxylum* species and preliminary insight into the antibiofilm potential of their sesquiterpenoids, particularly for compounds with limited previous antibiofilm reports.

## 1. Introduction

Sesquiterpenoids are the most abundant class of terpenoids, biosynthetically derived from three isoprene units. As of 2014, more than 10,000 sesquiterpenoids with diverse and structurally intriguing scaffolds had been reported [1]. These compounds play essential roles in various sectors, particularly as major constituents of fragrances along with monoterpenoids [2,3]. In recent years, sesquiterpenoids have also attracted considerable attention due to their broad range of pharmacological activities, including neuroprotective, anti-inflammatory, antidepressant, anti-hepatic fibrosis, and antibacterial effects [4,5,6,7]. Notably, several newly identified sesquiterpenoids from natural sources have demonstrated significant antibacterial activity against pathogenic bacteria such as *Staphylococcus aureus*, methicillin-resistant *Staphylococcus aureus* (MRSA), *Bacillus* spp., *Erwinia carotovora*, and *Pseudomonas* spp. [8,9]. In addition to their antibacterial properties, sesquiterpenoid-rich essential oils containing compounds such as spathulenol and *β*-caryophyllene oxide have been reported to exhibit significant antibiofilm activity against biofilm-forming microorganisms, including *Streptococcus mutans*. Essential oils rich in spathulenol were shown to inhibit *S. mutans* biofilm formation by up to 80%, while β-caryophyllene oxide-containing oils also demonstrated inhibitory effects against *S. mutans* biofilms [10,11].

The reported antibiofilm potential of sesquiterpenoids has encouraged further investigation into their possible applications against biofilm-associated infections. Biofilms are structured microbial communities embedded within a self-produced extracellular matrix that enhances bacterial persistence and resistance to antimicrobial agents [12]. They are estimated to be responsible for approximately 65–80% of chronic infections in the human body and have been detected in nearly all organ systems, including the digestive and excretory systems. Biofilm-associated diseases include acute pneumonia, chronic rhinosinusitis, chronic otitis media, urinary tract infections, chronic prostatitis, bacterial vaginosis, osteomyelitis, atherosclerosis, dental caries, gastric ulcers, and inflammatory bowel disease [13,14]. Both Gram-positive and Gram-negative bacteria are capable of forming biofilms; however, *Streptococcus mutans* is among the most prevalent and clinically relevant biofilm-forming bacteria. *S. mutans* plays a central role in the initiation and progression of dental caries through its strong adhesion to tooth surfaces, its acidogenic and aciduric properties, and its ability to synthesize extracellular polysaccharides that stabilize biofilm architecture [15]. Consequently, inhibition of *S. mutans* biofilm formation is considered a key strategy for the prevention and management of dental caries, a disease with a reported global prevalence of up to 82.8% [14].

The Meliaceae family, a member of the order Sapindales, comprises tropical plant species that are widely recognized for their valuable timber and fragrant stems. It includes approximately 58 genera with around 740 species [16]. Sesquiterpenoids have been reported from several genera within this family, including *Aglaia* [17], *Dysoxylum* [18], *Chisocheton* [19], *Guarea* [20], *Trichilia* [21], and *Lansium* [22]. The genus *Dysoxylum* exhibits a broad geographical distribution, mainly across tropical and subtropical areas such as China, India, Malaysia, northeastern Australia, and other regions of Southeast Asia. To date, about 200 species of *Dysoxylum* have been identified as sources of diverse secondary metabolites, including sesquiterpenoids [23,24], sesquiterpenoid dimers [25], diterpenoids [26,27], triterpenoids [28,29,30,31], limonoids [32,33,34], and macrolides [35]. Previous phytochemical investigations on *D. acutangulum* have reported the presence of sesquiterpenoids [36], alkaloids [37,38,39], and triterpenoids [40] exhibiting cytotoxic, tyrosinase inhibitory, and osteoclast differentiation inhibitory activities, whereas studies on *D. cauliflorum* have described the presence of essential oils [41] and triterpenoids [42]. Nevertheless, studies specifically focusing on the sesquiterpenoid constituents and their antibiofilm potential, particularly against *S. mutans*, remain limited.

Previous studies reported the isolation of phenolic sesquiterpenoids, including dioxophenol and (7*R*,10*S*)-2-hydroxycalamenene, from the twigs of *D. densiflorum*, both of which exhibited antibacterial activity against *Bacillus subtilis* with minimum inhibitory concentration (MIC) values of 28 μM [43]. Motivated by these findings, we investigated the sesquiterpenoid constituents of the less studied species *D. acutangulum* Miq. and *D. cauliflorum* Hiern. In this study, four sesquiterpenoids representing diverse scaffolds were isolated: spathulenol (**1**), an isodaucane-type sesquiterpenoid obtained as an epimeric mixture consisting of 10-oxo-isodauc-3-en-15-al (**2a**) and sinulin A (**2b**), *β*-caryophyllene oxide (**3**), and humulene diepoxide A (**4**). Although compounds **1**, **3**, and **4** are widely distributed sesquiterpenoids previously reported from various plant species [16], their occurrence in *D. acutangulum* (**1** and **3**) and *D. cauliflorum* (**4**) has not previously been reported based on available phytochemical studies of these species [36,37,38,39,40,41,42]. Furthermore, to the best of our knowledge, compounds **2a** and **2b** have not previously been described from the genus *Dysoxylum* based on currently available phytochemical reports of the genus [16].

Their isolation and structural elucidation, along with in vitro and in silico interaction studies against biofilm-associated targets against *S. mutans*, are reported herein. This study highlights the potential of naturally derived compounds as promising antibiofilm agents, supporting the growing interest in nature-based approaches for bioactive compound discovery.

## 2. Results and Discussion

### 2.1. Isolation and Structural Elucidation

**Figure 1:**
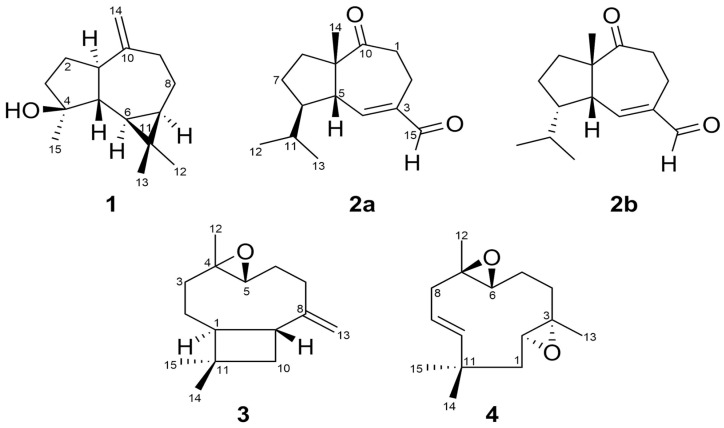
The structure of compounds **1**–**4**.

The ethanolic extract from the twigs of *D. acutangulum* Miq. and *D. cauliflorum* Hiern. was macerated and extracted consecutively with *n*-hexane, ethyl acetate, and *n*-butanol. The *n*-hexane extract of *D. acutangulum* and *D. cauliflorum* was separated by a combination of normal-phase and reversed-phase column chromatography to give compounds **1**, **2**, **3**, and **4** as shown in Figure 1.

Compound **1** was isolated as a colorless oil, with the molecular formula C_15_H_24_O based on HR-TOFMS with the positive ion peak *m*/*z* 221.1918 [M + H]^+^ (calcd. 221.1905) (Figure S1), resulting in four degrees of unsaturation. IR spectra (Figure S2) showed absorption bands indicating the presence of hydroxyl (3379 cm^−1^), C-H *sp^3^* aliphatic (2927 cm^−1^), C=C olefinic (1635 cm^−1^), *gem*-dimethyl (1455 and 1375 cm^−1^), and an ether group (1095 cm^−1^). The ^1^H-NMR spectra (Figure S3) showed proton resonance related to three tertiary methyls at *δ*_H_ 1.04 (3H, s, CH_3_-12), 1.06 (3H, s, CH_3_-13), and 1.28 (3H, s, CH_3_-15); olefinic methylene at *δ*_H_ 4.66 (1H, br.s, H-14) and 4.69 (1H, br.s, H-14); and two methines at *δ*_H_ 0.46 (1H, dd, *J* = 9.5, 11.3 Hz, H-6) and 0.71 (1H, ddd, *J* = 11.3, 9.4, 6.1 Hz, H-7). Analysis of ^13^C-NMR supported by DEPT 135° spectra of compound **1** (Figure S4) showed 15 carbon resonances consisting of three methyls at *δ*_c_ [16.3 (C-12), 26.1 (C-15), 28.6 (C-13)], one aliphatic quaternary carbon *δ*_c_ [20.3 (C-11)], four aliphatic methylenes *δ*_c_ [24.8 (C-8), 26.7 (C-2), 38.8 (C-9), 41.7 (C-3)], four aliphatic methines *δ*_c_ [27.5 (C7), 29.9 (C-6), 53.4 (C-1), 54.3 (C-5)], one oxygenated quaternary carbon *δ*_c_ [81.0 (C-4)], one olefinic methylene *δ*_c_ [106.2 (C-14)], and one olefinic quaternary carbon *δ*_c_ [153.4 (C-10)]. Based on ^1^H-NMR, ^13^C-NMR, and DEPT 135°, compound **1** had four unsaturated degrees. One unsaturation degree came from the terminal olefinic group (C=CH_2_), while the remaining ones came from the tricyclic framework. The upfield proton signals in the ^1^H NMR spectrum at *δ*_H_ 0.46 (1H, dd, *J* = 11.3, 9.5, 6.1 Hz, H-6) and 0.71 (1H, ddd, *J* = 11.3, 9.4, 6.1 Hz, H-7) suggested the presence of a cyclopropane moiety of a sesquiterpenoid aromadendrane framework, which was supported by the existence of an upfield quaternary carbon at *δ*_c_ 20.3 (C-11) [44]. Comparison of the NMR data of **1** (Table 1) with those of previously reported compounds revealed close agreement with spathulenol [45]. The optical rotation (${\left[\alpha \right]}_{D}^{26}$ +1.25° (c 0.001, CHCl_3_)) showed agreement with (+)-spathulenol, supporting the assigned stereochemistry of **1** [46].

Compound **2** was isolated as an epimeric mixture, which was obtained as a yellowish oil. On the basis of the ^1^H-NMR (Figure S6) and ^13^C-NMR spectra (Figure S7), the ratio of the mixture of **2a** and **2b** was deduced as 4:1. The assignment of **2a** as the major component and **2b** as the minor component was based on the relative intensities of the corresponding proton and carbon resonances in the ^1^H and ^13^C NMR spectra, along with their respective 2D-NMR correlations. Most of the signals were well resolved for both compounds. Compound **2** yielded a molecular formula of C_15_H_22_O_2_, as determined by HR-TOFMS, with an *m*/*z* value of 257.1520 [M + Na]^+^ (calcd. 257.1517) (Figure S5), indicating five unsaturation degrees. Furthermore, the ^1^H-NMR spectra of the major compound **2a** presented one methyl singlet resonance at *δ*_H_ 1.32 (3H, s, CH_3_-14) and two doublet methyl resonances at *δ*_H_ 0.94 (3H, d, CH_3_-13) and 0.93 (3H, d, CH_3_-12). These methyls had the same *J* value, 6.8 Hz. Additionally, one methine *sp^2^* at *δ*_H_ 6.63 (1H, d, *J* = 5.5 Hz, H-4) and an aldehyde proton resonance at *δ*_H_ 9.30 (1H, s, H-15) were also present in the ^1^H-NMR spectrum. The ^13^C-NMR spectrum accompanied by DEPT 135º of **2a** showed the presence of 15 carbons, including three methyl resonances at *δ*_c_ 19.7 (C-13), 22.0 (C-12), and 25.0 (C-14); four methylenes resonance at *δ*_c_ 38.9 (C-1), 19.7 (C-2), 26.8 (C-7), and 35.2 (C-8); four methines at *δ*_c_ 53.2 (C5), 55.4 (C-6), 32.4 (C-11) and an olefinic methine at *δ*_c_ 158.7 (C-4); and three quaternary carbon resonances at *δ*_c_ 59.7 (C-9), including an olefinic quaternary carbon resonance at *δ*_c_ 143.8 (C-3) and a carbonyl ketone at *δ*_c_ 212.2 (C-10), as well as one aldehyde at *δ*_c_ 192.8 (C-15). Based on ^1^H-NMR, ^13^C-NMR, and DEPT 135°, compound **2a** had a methine olefinic group (C=CH), a ketone, and an aldehyde. Therefore, the two remaining unsaturation degrees were attributed to the bicyclic system of a sesquiterpenoid. The presence of one quaternary aliphatic carbon, one tertiary methyl, and two secondary methyl carbons indicates the characteristic of isodaucane-type sesquiterpenoid [47].

**Figure 2:**
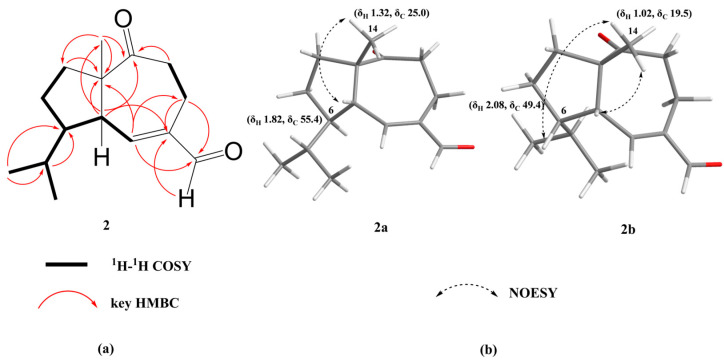
Selected ^1^H−^1^H COSY and HMBC correlations of **2** (**a**) and NOESY correlations of **2a** and **2b** with colors of the molecular model: gray = carbon, white = hydrogen, and red = oxygen (**b**).

The ^1^H-NMR and ^13^C-NMR of **2a** and **2b** showed high similarity, supporting the same planar structure, with some chemical shift differences arising from stereochemical differences (Table 2). To further confirm the structure of this mixture, comprehensive 2D NMR experiments were performed (Figures S8–S11). The ^1^H−^1^H COSY spectrum established the isodaucane skeleton, including the endocyclic double bond, as evidenced by the correlation between H-5 and the olefinic proton H-4. HMBC correlations were then used to assign the positions of the functional groups. The tertiary methyl at *δ*_H_ 1.32 (H-14) showed correlation with the quaternary carbon C-9 and the ketone at C-10, suggesting a neighboring position. In addition, the olefinic proton at *δ*_H_ 6.63 (H-4) showed correlation with the aldehyde (C-15), a methylene at C-2, and a quaternary at C-9. Based on these correlations, the planar structures of **2a** and **2b** were established, as shown in Figure 2. The NOESY experiment was conducted to further confirm the stereochemistry of both the major and minor components. H-14, a methyl group assigned in the *β*-orientation based on isodaucane scaffold, was used as a reference to deduce the configurations of the remaining stereocenters [48,49]. In the major component **2a**, a NOESY correlation between H-14 and H-6 indicated a *β*-orientation for H-6. In contrast, compound **2b** showed NOESY correlations of H-14 with both H-6 and H-5, suggesting a difference at one stereogenic center and indicating that **2a** and **2b** constituted an epimeric mixture. Comparison of the NMR data of **2a** and **2b** with those of previously reported compounds revealed close agreement with 10-oxo-isodauc-3-en-15-al and sinulin A, respectively [48,50]. Accordingly, compounds **2a** and **2b** were identified as a mixture of 10-oxo-isodauc-3-en-15-al and sinulin A, isolated for the first time from *Dysoxylum* genus.

Compound **3** was obtained as a colorless oil. Its molecular formula (C_15_H_24_O) was established by HR-TOFMS based on the positive ion peak at *m*/*z* 221.1910 [M + H]^+^ (calcd. 221.1905) (Figure S12), corresponding to four degrees of unsaturation. The IR spectrum (Figure S13) showed absorption bands characteristic of aliphatic sp^3^ C–H (2931 cm^−1^), olefinic C=C (1677 cm^−1^), *gem*-dimethyl groups (1454 and 1383 cm^−1^), and an ether functionality (1074 cm^−1^). The ^1^H NMR spectrum (Figure S14) displayed three methyl singlets at δ_H_ 0.98 (3H, s, CH_3_-15), 1.00 (3H, s, CH_3_-14), and 1.19 (3H, s, CH_3_-12). In addition, signals at δ_H_ 4.85 (1H, s, H-13a) and 4.97 (1H, s, H-13b) were assigned to an olefinic methylene group. Analysis of the ^13^C NMR and DEPT-135 spectra (Figure S15) revealed 15 carbon signals, consisting of three methyls [δ_C_ 17.0 (C-12), 21.6 (C-14), 29.9 (C-15)], five methylenes [δ_C_ 27.2 (C-2), 29.8 (C-6), 30.2 (C-7), 39.1 (C-3), 39.8 (C-10)], three methines [δ_C_ 48.7 (C-9), 50.7 (C-1), 63.8 (C-5)], and two quaternary carbons [δ_C_ 34.0 (C-11) and 59.8 (C-4)], along with one olefinic methylene [δ_C_ 112.8 (C-13)] and one olefinic quaternary carbon [δ_C_ 151.8 (C-8)]. These data indicated the presence of one olefinic methylene (C=CH_2_), accounting for one degree of unsaturation. The remaining three degrees of unsaturation suggested a tricyclic sesquiterpenoid framework. One of the rings was suggested to contain an epoxide moiety, supported by the characteristic ^1^H NMR signal at δ_H_ 2.87 and the corresponding ^13^C shifts at δ_C_ 63.8 (C-5, methine) and 59.8 (C-4, quaternary carbon). The overall NMR features were consistent with a caryophyllene-type skeleton [51], particularly the presence of three methyl singlets and a *gem*-dimethyl group (CH_3_-14 and CH_3_-15). Comparison of the NMR data of **3** (Table 3) with reported values for β-caryophyllene oxide isolated from *Dysoxylum* showed close agreement [51]. Therefore, compound **3** was identified as β-caryophyllene oxide.

Compound **4** was isolated as a white amorphous solid. Its molecular formula was determined to be C_15_H_24_O_2_ by HR-TOFMS, which showed a protonated molecular ion peak at *m*/*z* 237.1844 [M + H]^+^ (calcd. 237.1855) (Figure S16), consistent with four degrees of unsaturation. The IR spectrum (Figure S17) exhibited absorption bands for aliphatic C–H (2960–2936 cm^−1^), olefinic C=C (1711–1684 cm^−1^), *gem*-dimethyl groups (1468 and 1389 cm^−1^), and C–O (1078 cm^−1^). The absence of a broad O–H band suggested that the oxygen atoms were not present as free hydroxyl groups [52]. The ^1^H-NMR spectrum (Figure S18) showed characteristics of a sesquiterpenoid skeleton, with most proton resonances appearing in the δ_H_ 1.0–2.0 ppm region. Four tertiary methyl groups were observed at δ_H_ 1.08 (3H, s, H-13), 1.20 (3H, s, H-12), and 1.30 (6H, s, H-14 and H-15), indicating a *gem*-dimethyl moiety. Two olefinic protons at δ_H_ 5.49 (1H, ddd, *J* = 15.7, 10.7, 5.0 Hz, H-9) and 5.32 (1H, d, *J* = 15.6 Hz, H-10) suggested a *trans*-disubstituted double bond, supported by the large coupling constants (*J* > 15 Hz). In addition, two oxygenated methine protons were detected at δ_H_ 2.48 (1H, d, *J* = 9.6 Hz, H-2) and 2.73 (1H, dd, *J* = 10.1, 5.0 Hz, H-6), appearing relatively upfield due to shielding effects associated with epoxide functionalities [52]. 

The ^13^C-NMR spectrum, supported by DEPT-135 analysis (Figure S19), revealed 15 carbon signals consisting of four methyls, four methylenes, two olefinic methines, two oxygenated methines, and three quaternary carbons. Notably, four oxygenated carbons observed at δ_C_ 64.7 (C-2), 60.3 (C-6), 60.1 (C-3), and 63.4 (C-7) fell within the characteristic range for epoxide carbons (δ_C_ 58–62 ppm), whereas hydroxylated carbons typically resonated more downfield (δ_C_ 73–75 ppm) [52]. The olefinic carbons at δ_C_ 122.6 (C-9) and 142.9 (C-10) confirmed the presence of one double bond. Based on the spectroscopic data, the four degrees of unsaturation were attributed to one double bond and three rings, including two epoxide-containing rings within a monocyclic sesquiterpenoid framework. The presence of multiple methyl groups, a *gem*-dimethyl unit, and the overall carbon distribution suggested a humulene-derived sesquiterpenoid framework rather than bisabolane, germacrane, or elemane frameworks. From a biosynthetic perspective, such a structure can arise from cyclization of a farnesyl cation to a humulyl cation, followed by oxidative transformations such as epoxidation [53]. Accordingly, the double bond was assigned at C-9/C-10, while the epoxide moieties were located at C-2/C-3 and C-6/C-7. Comparison of the NMR data for compound **4** (Table 3) with those reported in the literature showed close agreement with humulene diepoxide A [51]. Furthermore, the optical rotation value, ${\left[\alpha \right]}_{D}^{26}$ −6.55° (c 0.1, CHCl_3_), was consistent with the reported value, confirming the stereochemistry of compound **4** [52]. Therefore, compound **4** was identified as humulene diepoxide A.

### 2.2. Antibiofilm Activity

To the best of our knowledge, antibiofilm activity against *S. mutans* has not previously been reported for these sesquiterpenoids isolated from *D. acutangulum* and *D. cauliflorum*. The present findings therefore expand the chemical and biological relevance of sesquiterpenoids from the genus *Dysoxylum*, particularly in the context of oral biofilm inhibition.

Table 4 summarizes the antibiofilm activity of the *n*-hexane extract, isolated compounds **1**–**4**, and chlorhexidine against *Streptococcus mutans* ATCC 25175, expressed as minimum biofilm inhibitory concentration (MBIC). The *n*-hexane extract exhibited relatively weak activity, with an MBIC value of 2500 µg/mL. In contrast, the isolated compounds demonstrated improved antibiofilm potency. Compound **1** inhibited biofilm formation at 500 µg/mL with a percentage inhibition of 53.9%, while compound **2** showed an MBIC value of 250 µg/mL but with a lower inhibition percentage of 36.1%, indicating that biofilm suppression at this concentration was not yet optimal. Compound **3** showed no inhibitory activity (NI), whereas compound **4** exhibited an MBIC of 250 µg/mL with 53.6% inhibition, comparable to compound **1** in terms of efficacy. Although chlorhexidine remained the most active reference agent (MBIC 62.5 µg/mL), the enhanced activity observed for the purified compounds compared to the crude extract clearly indicated that antibiofilm efficacy was concentrated within specific chemical constituents rather than the extract as a whole. These findings underscore that a compound-oriented approach in the search for antibiofilm agents may provide clearer insight into the active constituents responsible for antibiofilm activity compared to crude extracts.

Previously, both (+)-spathulenol (**1**) and β-caryophyllene oxide (**3**) had been reported for their antibiofilm properties as essential oil constituents (26.21% and 8.80%, respectively), which showed significant activities [10,11]. In contrast, there is still very limited information regarding the antibiofilm properties of 10-oxo-isodauc-3-en-15-al (**2a**), sinulin A (**2b**), and humulene diepoxide A (**4**). Therefore, the observed activity of these compounds in the present study may provide new insight into the antibiofilm potential of these sesquiterpenoids.

Isolation of individual compounds enables more precise evaluation of their biological activity but also enables mechanistic elucidation at the molecular level, including target identification, binding interactions, and structure–activity relationship (SAR) analysis. Therefore, we conducted further investigations using in silico approaches to provide deeper insight into molecular interactions of sesquiterpenoid-based compounds with biofilm-associated targets and to rationalize their in vitro inhibitory profiles.

### 2.3. Molecular Docking Result

The molecular docking simulations provided significant insights into the binding preferences of the test ligands—**1**, **2a**, **2b**, **3**, and **4**—compared to the reference antibiofilm agent chlorhexidine against three key virulence targets of *Streptococcus mutans*. The binding affinities and the nature of the chemical interactions are summarized in Table 5.

**Figure 3:**
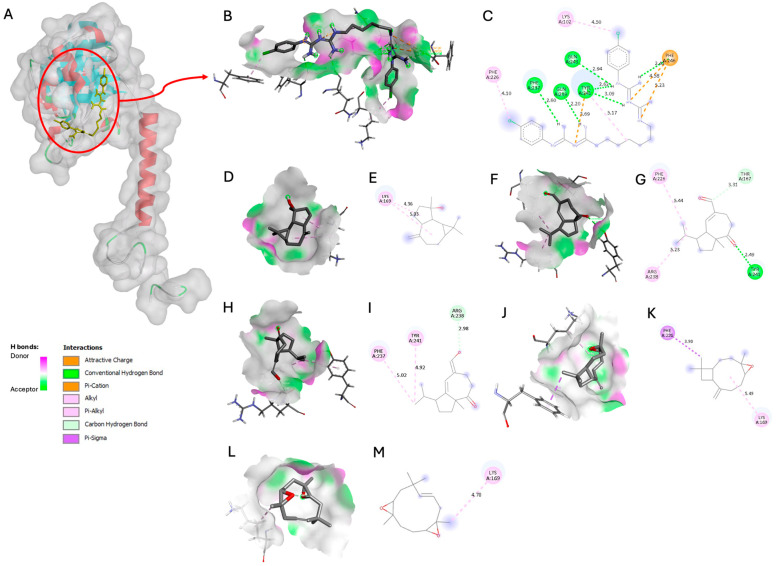
Molecular docking analysis showing the 3D binding modes and 2D interaction profiles of chlorhexidine and the isolated compounds (**1**, **2a**, **2b**, **3**, and **4**) within the active site of *Streptococcus mutans* Sortase A (SrtA). (**A**) Full view of the Sortase A protein surface and binding pocket indicated by the red circle; (**B**) close-up 3D view of chlorhexidine in the binding site; (**C**) 2D interaction diagram for chlorhexidine; (**D**,**F**,**H**,**J**,**L**) 3D binding poses of **1**, **2a**, **2b**, **3** and **4**; (**E**,**G**,**I**,**K**,**M**) corresponding 2D interaction maps for the ligands in (**D**,**F**,**H**,**J**,**L**). Stick representations (3D view) of interacting amino acid residues and the ligand, with atoms color-coded as follows: gray = carbon, white = hydrogen, red = oxygen, and blue = nitrogen. For the 2D view, each interaction is coded as follows: Hydrogen bonding interactions are shown in dark green (conventional hydrogen bonds) and light green (non-conventional hydrogen bonds including carbon-hydrogen bonds and π-donor hydrogen bonds). Hydrophobic interactions are depicted in light pink (alkyl and π-alkyl contacts). Aromatic interactions are shown in purple (π-σ interactions). Electrostatic interactions are represented in orange (salt bridges, π-cation interactions, and attractive charges). Amino acid residues are labeled, and interaction distances are indicated in Ångströms (Å).

**Figure 4:**
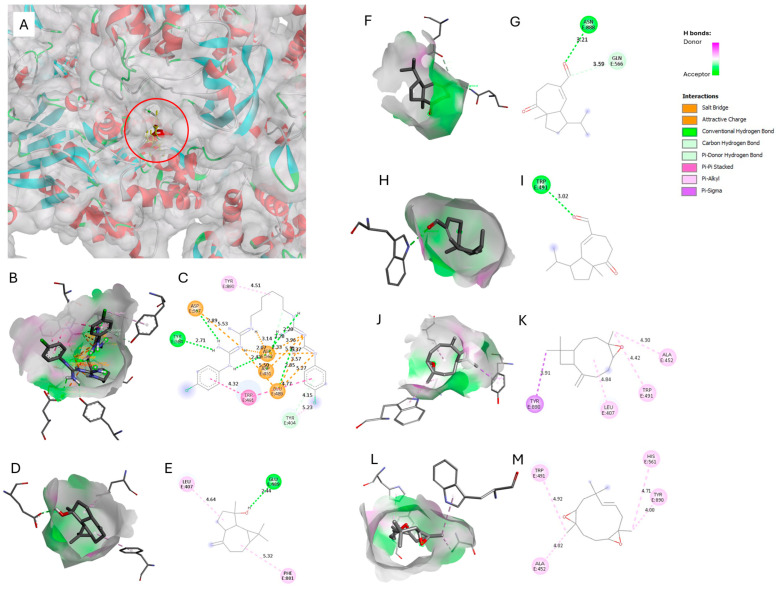
Molecular docking analysis showing the 3D binding modes and 2D interaction profiles of chlorhexidine and the isolated compounds (**1**, **2a**, **2b**, **3**, and **4**) within the active site of *Streptococcus mutans* GtfB. (**A**) View of binding pocket of GtfB indicated by the red circle; (**B**) close-up 3D view of chlorhexidine in the binding site; (**C**) 2D interaction diagram for chlorhexidine; (**D**,**F**,**H**,**J**,**L**) 3D binding poses of **1**, **2a**, **2b**, **3**, and **4**; (**E**,**G**,**I**,**K**,**M**) corresponding 2D interaction maps for the ligands in (**D**,**F**,**H**,**J**,**L**). Stick representations (3D view) of interacting amino acid residues and the ligand, with atoms color-coded as follows: gray = carbon, white = hydrogen, red = oxygen, and blue = nitrogen. For the 2D view, each interaction is coded as follows: Hydrogen bonding interactions are shown in dark green (conventional hydrogen bonds) and light green (non-conventional hydrogen bonds, including carbon-hydrogen bonds and π-donor hydrogen bonds). Hydrophobic interactions are depicted in light pink (alkyl and π-alkyl contacts) and pink (π-π stacked interactions). Aromatic interactions are shown in purple (π-σ interactions). Electrostatic interactions are represented in orange (salt bridges, π-cation interactions, and attractive charges). Amino acid residues are labeled, and interaction distances are indicated in Ångströms (Å).

Across the targeted receptors, chlorhexidine exhibited the strongest binding affinities for Sortase A (−7.931 kcal/mol) and GtfB (−8.309 kcal/mol). The superior stability of chlorhexidine with the *S. mutans* receptor complex was primarily driven by a dense network of polar interactions, specifically conventional hydrogen bonds with residues such as Glu489, Asp562, and Asp567, further stabilized by Pi–Pi-stacked non-polar interactions, as shown in Figure 3 and Figure 4. In contrast, the test ligands primarily leveraged hydrophobic forces, including Pi–alkyl, alkyl, and Pi–sigma interactions, with a lower frequency of hydrogen bonding. Within the Sortase A (SrtA) active site, as shown in Figure 3, ligand **2a** formed polar interactions with Tyr241 and Thr167, which likely resulted in a higher affinity (−6.266 kcal/mol) compared to Spathulenol (−6.072 kcal/mol). However, the highest binding affinity for Sortase A was observed for ligand **3** (−6.620 kcal/mol), which was primarily mediated through non-polar interactions. Notably, the binding scores for ligand **2a** and **4** were nearly identical, mirroring the experimental MBIC test results of 250 µg/mL. This trend persisted in Glucosyltransferase B, where chlorhexidine remained the most potent inhibitor, followed by ligands **2** and **4**, with both maintaining consistent affinities near −7 kcal/mol.

Interestingly, compound **3** exhibited relatively favorable docking scores despite showing no detectable antibiofilm activity in vitro. This discrepancy may reflect limitations of docking simulations, which do not account for factors such as solubility, membrane permeability, molecular stability, or bioavailability under experimental conditions.

The computational analysis revealed that the test ligands exhibited moderate-to-high binding affinities toward the targeted *S. mutans* virulence proteins. Notably, the chlorhexidine, the control drug, demonstrated superior theoretical affinity for SortA and GtfB compared to all ligands, suggesting significant potential for disrupting the initial stages of biofilm scaffolding. These in silico observations provided a strong foundation for the subsequent in vitro MBIC evaluations (Table 4). The observed rank-order correlation validated the discriminatory power of the docking model, as it accurately categorized the potency profiles of the control and test compounds. Furthermore, the data aligned with the thermodynamic principles described by a previously reported method [54], in which a ∆G variance of approximately 1.36 kcal/mol corresponds to a tenfold shift in binding magnitude. The transition from −8 to −6 kcal/mol represents a 20- to 30-fold difference in theoretical affinity, which is biologically consistent with the 4- to 8-fold increase observed in the experimental MBIC values in SrtA and GtfB enzymes.

Consistent with the bioassay evaluations (MBIC test) of the isolated sesquiterpenoids (compounds **1**, **2a**, **2b**, **3**, and **4**), the high degree of concordance between the in silico docking data (Table 5) and the in vitro MBIC results (Table 4) further reinforces the predictive validity of the computational model. Although compound **1** theoretically displayed comparable or slightly favorable binding energies toward SrtA and GtfB relative to **2b** (−6.072 vs. −6.072 kcal/mol in SrtA; −7.166 vs. −6.949 kcal/mol in GtfB), its in vitro antibiofilm activity (MBIC 500 µg/mL) was inferior to compound **2** and **4** (MBIC 250 µg/mL). This apparent discrepancy may indicate that factors beyond docking affinity, such as physicochemical properties, membrane permeability, or possible interactions between constituents in the epimeric mixture, could have contributed to the observed antibiofilm activity. Such interaction often yields enhanced biological efficacy compared to a single sesquiterpenoid entity.

Synergism and sensitization phenomena have been widely reported for sesquiterpenes; similar modulatory effects have been reported for other sesquiterpenoids, including nerolidol isomers, which enhanced antibacterial activity through membrane-related mechanisms [55,56,57]. Similarly, the combined presence of **2a**, **2b**, and **4** may facilitate improved target accessibility, membrane interaction, or multi-site engagement within *S. mutans* virulence enzymes, thereby amplifying antibiofilm performance beyond what is predicted solely from individual docking scores. Therefore, while docking accurately discriminated against the general potency hierarchy (chlorhexidine > test ligands), the biological outcome underscores the importance of compositional and cooperative effects in sesquiterpenoid-based antibiofilm agents. Collectively, these findings not only demonstrate alignment between thermodynamic predictions and experimental MBIC trends but also provide new insight into the potential of sesquiterpenoid mixtures as promising antibiofilm scaffolds, warranting further mechanistic and combination-based investigations.

## 3. Materials and Methods

### 3.1. Materials

Analytical-grade solvents, including methanol, ethyl acetate, n-hexane, methylene chloride, chloroform, and distilled water, were purchased from Merck KGaA, Darmstadt, Germany and used throughout the experiments. Vacuum liquid chromatography (VLC) and column chromatography (CC) were performed using silica gel 60 (70–230 and 230–400 mesh; Merck KGaA, Darmstadt, Germany) and octadecyl silane (ODS, Chromatorex C18 DM1020T, 100–200 mesh; Fuji Silysia Chemical Ltd., Kasugai, Japan). Fractions were monitored by thin-layer chromatography (TLC) on normal-phase silica gel 60 F254 and reversed-phase RP-18 F254S plates (Merck KGaA, Darmstadt, Germany). TLC spots were visualized under UV light at 254 and 365 nm, followed by spraying with 10% H_2_SO_4_ in ethanol and heating. *Streptococcus mutans* ATCC 25175 was used for the antibiofilm activity assay.

### 3.2. Plant Material

Twigs of *Dysoxylum acutangulum* Miq. and *Dysoxylum cauliflorum* Hiern were collected from the Bogor Botanical Gardens, Indonesia. The plant materials were identified at Herbarium Bogoriense, and voucher specimens were deposited under collection numbers III.F.20 (*D. acutangulum*) and III.D.16 (*D. cauliflorum*). The twigs of *D. acutangulum* and *D. cauliflorum* were cut into small pieces, air-dried in a shaded and well-ventilated room at ambient temperature for approximately one week, and subsequently pulverized into a fine powder using a mechanical grinder before extraction.

### 3.3. Extraction and Isolation

Dried twig powder of *D. acutangulum* Miq. (5.6 kg) was macerated with 96% ethanol (20 L) at room temperature. The resulting macerate was filtered and concentrated under reduced pressure using a rotary evaporator at 40 °C to afford a crude ethanolic extract (340.9 g). The crude extract was subsequently suspended and subjected to successive liquid–liquid partitioning based on solvent polarity using *n*-hexane, ethyl acetate, and *n*-butanol. Each fraction was concentrated under vacuum to yield *n*-hexane extract (37.5 g), ethyl acetate extract (113 g), and *n*-butanol extract (1 g), respectively.

The *n*-hexane fractions were prioritized for further chromatographic separation based on their TLC profiles, which indicated the presence of abundant terpenoid constituents. In addition, the isolation strategy was designed sequentially according to compound polarity, beginning with non-polar to semi-polar fractions to facilitate systematic separation of sesquiterpenoid constituents. The *n*-hexane extract (37.5 g) was separated on vacuum liquid chromatography (VLC) with a stepwise gradient of *n*-hexane: EtOAc: MeOH (100:0-0:100-100:0, 10% *v*/*v*) to yield 11 fractions (Fr. A–K). Fr. C (6.3 g) was chromatographed on silica gel CC using a stepwise gradient of *n*-hexane:EtOAc (100:0-0:100, 1% *v*/*v*) to yield 10 subfractions (Fr. C1–C10). Fr. C3 (117 mg) was subjected to ODS CC using MeOH-H_2_O (8:2) to yield 2 fractions (Fr. C3.1–C3.2). Fr. C3.2 (12 mg) was subjected to CC silica gel with n-hexane:DCM (7:3) to afford **3** (5 mg). Fr. C6 (306 mg) was subjected to ODS CC using MeOH:H_2_O (8:2) to yield 7 subfractions (Fr. C6.1–C6.7). Fr. C6.5 (16 mg) was subjected to CC silica gel with *n*-hexane:DCM (6:4) to afford **1** (10 mg). Fr. C8 (638 mg) was subjected to CC silica gel with *n*-hexane:DCM (5:5) to yield 4 subfractions (Fr. C8.1–C8.4). Subsequently, Fr. C8.3 (45 mg) was subjected to ODS CC using MeOH:H_2_O (6:4) to afford an epimeric mixture of **2a** and **2b** (7 mg).

Dried twig powder of *D. cauliflorum* Hiern (6.4 kg) was macerated using the same method to afford a crude ethanolic extract (466.6 g). The crude extract was suspended in EtOH–H_2_O (7:3) and successively partitioned with *n*-hexane, ethyl acetate, and *n*-butanol. Each fraction was concentrated under vacuum to yield *n*-hexane (38.1 g), ethyl acetate (72.4 g), and *n*-butanol (2.0 g) extracts, respectively. The *n*-hexane extract (38.1 g) was subjected to vacuum liquid chromatography (VLC) on silica gel using a stepwise gradient of *n*-hexane–EtOAc followed by EtOAc–MeOH (10% *v*/*v*) to afford ten fractions (Fr. A–J). Fr. C (14.2 g) was further separated by silica gel column chromatography using a gradient of *n*-hexane–EtOAc to yield seven subfractions (Fr. C1–C7). Fr. C2 (7.0 g) was repeatedly chromatographed on silica gel using gradient systems of *n*-hexane–EtOAc to afford seven subfractions (Fr. C2a–C2g). Further purification of Fr. C2c (1.1 g) by silica gel column chromatography (230–400 mesh) yielded eleven subfractions (Fr. C2c1–C2c11). Fr. C2c8 (119 mg) was subsequently purified by reversed-phase ODS column chromatography using MeOH–H_2_O (6:4), followed by silica gel column chromatography with *n*-hexane–CH_2_Cl_2_, to afford compound **4** (15.4 mg).

### 3.4. Compound Characterization

Spathulenol (**1**): Colorless oil, IR (KBr) ν_max_ 3388; 2928, 1635, 1454, 1375, 1128 cm^−1^; ^1^H NMR (CDCl_3_, 700 MHz); ^13^C NMR (CDCl_3_, 175 MHz), see Table 1; HR-TOFMS *m*/*z* 221.1910 [M + H]^+^ (calculated for C_15_H_25_O, *m*/*z* 221.1905).

10-Oxo-isodauc-3-en-15-al (**2a**) and sinulin A (**2b**): Yellowish oil; ^1^H NMR (CDCl_3_, 700 MHz); ^13^C NMR (CDCl_3_, 175 MHz), see Table 2; HR-TOFMS *m*/*z* 257.1520 [M + Na]^+^ (calculated for C_15_H_22_O_2_Na, *m*/*z* 257.1517).

β-caryophyllene oxide (**3**): Colorless oil, IR (KBr) ν_max_ 2931, 1677, 1454, 1383, 1074 cm^−1^; ^1^H NMR (CDCl_3_, 700 MHz); ^13^C NMR (CDCl_3_, 175 MHz), see Table 3; HR-TOFMS *m*/*z* 221.1904 [M + H]^+^ (calculated for C_15_H_25_O, *m*/*z* 221.1905).

Humulene diepoxide A (**4**): Amorphous white solid; IR (KBr) ν_max_ 2960–2936, 1711–1684, 1468, 1389, 1078, 980 cm^−1^; ^1^H NMR (CDCl_3_, 700 MHz); ^13^C NMR (CDCl_3_, 175 MHz), see Table 3; HR-TOFMS *m*/*z* 237.1844 [M + H]^+^ (calculated for C_15_H_25_O_2_, *m*/*z* 237.1855).

### 3.5. Infrared Spectroscopy (IR)

IR spectra were recorded using a PerkinElmer Spectrum 100 FT-IR spectrometer (PerkinElmer, Waltham, MA, USA) with KBr discs (sample:KBr ratio = 1:100) in transmission mode over the range of 4000–400 cm^−1^. The spectra were processed and analyzed using Spectrum software 6.3.5.

### 3.6. Mass Spectroscopy (MS)

HR-TOF-ESI-MS spectra were acquired using a Waters XEVO HR-TOF-ESI-MS instrument (Milford, MA, USA). Electrospray ionization in positive-ion mode (ESI+) was used to generate the ions in the MS system. The obtained high-resolution *m*/*z* values were used to support molecular formula determination.

### 3.7. Nuclear Magnetic Resonance (NMR)

NMR spectra were recorded in CDCl_3_ using tetramethyl silane (TMS) as the internal standard on an Bruker AVANCE NEO 700 MHz spectrometer (Bruker, Billerica, MA, USA). ^1^H and ^13^C NMR spectra were measured at 700 and 175 MHz, respectively. Two-dimensional NMR experiments, including DEPT-135, ^1^H–^1^H COSY, HSQC, HMBC, and NOESY, were performed using standard pulse sequences.

### 3.8. Determination of Antibiofilm Activity

The antibiofilm activity was evaluated by determining the Minimum Biofilm Inhibitory Concentration (MBIC) using a crystal violet microdilution assay in a 96-well microplate. All equipment and materials were sterilized by autoclaving at 121 °C for 15 min prior to use. The test bacteria were grown on agar media at 37 °C for 18–24 h, and several colonies were suspended in Brain Heart Infusion Broth (BHIB) supplemented with 2% sucrose and incubated for a further 18–24 h. The bacterial suspension was adjusted to 0.5 McFarland standard (≈1–2 × 10^8^ CFU/mL) and dispensed into the microplate with appropriate controls, including sample control, negative control, solvent control, and positive control (chlorhexidine). After adding test samples and performing serial dilutions, the plate was incubated for 18–48 h. The wells were then washed with PBS, stained with 1% crystal violet, and the bound dye was solubilized with 30% glacial acetic acid. Biofilm formation was quantified by measuring absorbance at 595 nm using a multimode microplate reader (Infinite^®^ M200 Pro; Tecan Group Ltd., Männedorf, Switzerland). Biofilm percent inhibition was calculated by the following formula:

% biofilm inhibition = {(O.D. in control − O.D. of test)/O.D. in control} × 100(1)

### 3.9. Molecular Docking Analysis

To elucidate the theoretical binding interactions of compounds **1**–**4** against *Streptococcus mutans* enzymes involved in biofilm formation, such as Sortase A (SrtA) and glucotransferases (Gtfs), including GtfB, GtfC, and GtfD [58,59,60], molecular docking analysis was performed. The three-dimensional structures of all target protein structures for SrtA (4TQX) and GtfB (8FK4) were obtained from the RCSB Protein Data Bank. Protein preparation involved removing water molecules, adding polar hydrogens, and assigning Kollman charges using AutoDockTools 1.5.7 within a Python 3.9 environment in Jupyter Notebook 7.5.6. Moreover, three-dimensional ligand conformers were generated from 2D structures using RDKit in Python. This preparation included explicit hydrogen addition, 3D structure embedding via the Experimental Torsion-angle Knowledge Distance Geometry (ETKDGv3) algorithm, and structural relaxation using the Merck Molecular Force Field (MMFF94), with the Universal Force Field (UFF) utilized as a fallback for unsupported parameters. Molecular docking was executed using AutoDock Vina 1.2.7. An initial blind docking strategy was employed across the entire protein surface to identify potential binding pockets. The grid box used for each protein is described as follows: 4TQX coordinate at 18.0Å, 24.0Å, −3.0Å, and size at 40Å, 51Å, 70Å; 8FK4 coordinate at 24.0Å, 33.0Å, 118.0Å, and size at 70Å, 73Å, 118Å. Then, after tiling the grid box, the grid coordinate that produced highest binding affinity was chosen as follows: 4TQX coordinate at 23.0Å, 34.5Å, −8.0Å, and size at 30Å, 30Å, 30Å; 8FK4 coordinate at 44.0Å, 54.5Å, 74.0Å, and size at 30Å, 30Å, 30Å. The docking results were ranked based on the binding affinity scores (kcal/mol), and the binding interactions were visualized in BIOVIA, Dassault Systèmes, Discovery Studio, Release 2025, Dassault Systèmes (San Diego, CA, USA).

## 4. Conclusions

In conclusion, sesquiterpenoids were successfully isolated from the *n*-hexane extracts of *Dysoxylum acutangulum* Miq. and *D. cauliflorum* Hiern twigs. Spathulenol (**1**), 10-oxo-isodauc-3-en-15-al (**2a**) and sinulin A (**2b**) as an epimeric mixture, and β-caryophyllene oxide (**3**) were obtained from *D. acutangulum*, while humulene diepoxide A (**4**) was isolated from *D. cauliflorum*. All compounds are reported for the first time from their respective *Dysoxylum* species, whereas compounds **2a**–**2b** are reported for the first time from the *Dysoxylum* genus, providing additional chemotaxonomic insight into sesquiterpenoid diversity within the genus.

Compounds **1**, **2**, and **4** showed weak antibiofilm activity against *Streptococcus mutans*, with values ranging from 250 to 500 µg/mL, while compound **3** was inactive. Molecular docking results were consistent with the experimental data, showing moderate binding affinities toward Sortase A and Glucosyltransferase B. The epimeric mixture of compounds **2a**–**2b** also provided preliminary insight into possible stereochemical contributions to antibiofilm activity compared with single-component isolates.

Overall, this study represents the first in vitro antibiofilm and in silico evaluation of compounds **1**–**4** against *S. mutans* and expands the currently limited knowledge of sesquiterpenoid-based antibiofilm agents, particularly isodaucane-type sesquiterpenoids. Although the observed activities were moderate compared with chlorhexidine, the results provide preliminary insights into the antibiofilm potential of these sesquiterpenoids and may support future studies involving structural modification and mechanistic investigations.

## 5. Future Perspectives

Although compounds **1**, **2**, and **4** exhibited relatively weak antibiofilm activity against *Streptococcus mutans*, the present findings still provide preliminary insight into the antibiofilm potential of sesquiterpenoid scaffolds. Future studies should focus on structural modification and structure–activity relationship (SAR) analysis to identify the functional groups and stereochemical features contributing to antibiofilm activity, particularly in the epimeric mixture of compounds **2a** and **2b**. Such approaches may improve biological activity, selectivity, and physicochemical properties.

In addition, further mechanistic investigations are required to validate the molecular docking results and clarify the pathways involved in biofilm inhibition. Experimental studies targeting Sortase A and Glucosyltransferase B, as well as membrane interaction and quorum sensing-related mechanisms, would provide deeper understanding of the observed activity. Evaluation against resistant bacterial strains and more complex biofilm models may also help assess the broader relevance of these sesquiterpenoids as antibiofilm lead structures.

## Figures and Tables

**Table 1:** NMR data (700 MHz for ^1^H and 175 MHz for ^13^C, in CDCl_3_) for **1**.

No of C	Compound 1	
	δ_C_ (mult.)	δ_H_ (∑H, m, *J* (Hz))
1	53.4 (d)	1.30 (1H, m)
2	26.7 (t)	1.91 (1H, qd, 11.7; 6.3)
		1.63 (1H, m)
3	41.7 (t)	1.77 (1H, ddd, 12.8; 6.3; 1.5)
		1.57 (1H, m)
4	81.0 (s)	-
5	54.3 (d)	1.31 (1H, m)
6	29.9 (d)	0.46 (1H, dd, 9.5; 11.3)
7	27.5 (d)	0.71 (1H, ddd, 11.3; 9.4; 6.1)
8	24.8 (t)	1.98 (1H, dtd, 14.3; 6.2; 1.7)
		2.21 (1H, td, 10.5; 6.0)
9	38.8 (t)	2.42 (1H, dd, 6.3; 13.4)
		2.04 (1H, t, 13)
10	153.4 (s)	-
11	20.3 (s)	-
12	16.3 (q)	1.04 (3H, s)
13	28.6 (q)	1.06 (3H, s)
14	106.2 (t)	4.66 (1H, br.s)
		4.69 (1H, br.s)
15	26.1 (q)	1.28 (3H, s)

**Table 2:** NMR data (700 MHz for ^1^H and 175 MHz for ^13^C, in CDCl_3_) for **2a** and **2b**.

No of C	Compound 2a		Compound 2b		HMBC
	*δ*_C_ (mult.)	*δ*_H_ (∑H, m, *J* (Hz))	*δ*_C_ (mult.)	*δ*_H_ (∑H, m, *J* (Hz))	
1	38.9 (t)	2.79 (1Ha, dt, 14.1, 5.3)	38.0 (t)	2.56 (1H, m (overlap))	C2, C3, C10
		2.44 (1Hb, m)		2.43 (1H, m (overlap))	C1, C3, C4, C10, C15
2	19.7 (t)	2.72 (1H, dt, 14.6, 5.2)	20.1 (t)	3.00 (1H, ddd, 15.6, 7.1, 3.2)	-
		2.50 (1H, m)		2.05 (1H, m)	C2, C1, C5, C9, C3, C15
3	143.8 (s)	-	143.7 (s)	-	C14, C6, C9, C3, C4, C10
4	158.7 (d)	6.63 (1H, d, 5.5)	157.5 (d)	6.78 (1H, 5.0, 2.0)	C13, C9, C5, C7
5	53.2 (d)	2.53 (1H, m)	49.1 (d)	2.48 (1H, m (overlap))	C5, C9, C6, C8
6	55.4 (d)	1.82 (1H, m)	49.4 (d)	2.08 (1H, m)	C14, C5, C9, C7
7	26.8 (t)	1.44 (1H, m)	24.0 (t)	1.53 (1H, m)	-
		1.86 (1H, m)		1.91 (1H, m)	-
8	35.2 (t)	2.20 (1H, m)	33.3 (t)	2.24 (1H, m)	C7, C6
		1.41 (1H, m)		1.57 (1H, m)	C6, C11, C13
9	59.7 (s)	-	60.3 (s)	-	C6, C11, C12
10	212.2 (s)	-	213.3 (s)	-	C8, C5, C9, C10
11	32.4 (d)	1.65 (1H, m)	31.5 (d)	1.67 (1H, m)	C2, C3, C4
12	22.0 (q)	0.93 (3H, d, 6.8)	21.6 (q)	0.93 (3H, d, 6.9)	C2, C3, C10
13	19.5 (q)	0.94 (3H, d, 6.8)	18.9 (q)	0.88 (3H, d, 6.7)	C1, C3, C4, C10, C15
14	25.0 (q)	1.32 (3H, s)	19.5 (q)	1.02 (3H, s)	-
15	192.8 (d)	9.34 (1H, s)	192.8 (d)	9.49 (1H, s)	C2, C1, C5, C9, C3, C15

**Table 3:** NMR data (700 MHz for ^1^H and 175 MHz for ^13^C, in CDCl_3_) for **3** and **4**.

No of C	Compound 3		Compound 4	
	*δ*_C_ (mult.)	*δ*_H_ (∑H, m, *J* (Hz))	*δ*_C_ (mult.)	*δ*_H_ (∑H, m, *J* (Hz))
1	50.7 (d)	1.76 (1H, t, 9.8)	38.4 (t)	1.36 (1H, m)
2	27.2 (t)	1.63 (1H, m)	64.7 (d)	1.61 (1H, d, 14.2)
		1.61 (1H, m)		2.48 (1H, d, 9.6)
3	39.1 (t)	2.07 (1H, m)	60.1 (s)	-
		2.11 (1H, m)		
4	59.8 (s)	-	34.9 (t)	1.10 (1H, m)
				2.13 (1H, m)
5	63.8 (d)	2.87 (1H, dd, 4.5; 10.9)	25.2 (t)	1.40 (1H, m)
				2.20 (1H, m)
6	29.8 (t)	2.09 (1H, m)	60.3 (d)	0.73 (1H, dd, 10.5, 5.0)
		2.16 (1H, m)		
7	30.2 (t)	2.24 (1H, m)	63.4 (s)	-
		2.33 (1H, m)		
8	151.8 (s)	-	43.3 (t)	1.65 (1H, m)
				2.64 (1H, dd, 12.3, 5.0)
9	48.7 (d)	2.61 (1H, q, 9.6)	122.6 (d)	5.49 (1H, ddd, 15.7, 10,7, 5.0)
10	39.8 (t)	1.68 (1H, m)	142.9 (d)	5.32 (1H, d, 15.6)
		1.60 (1H, m)		
11	34.0 (s)	-	35.7 (s)	-
12	17.0 (q)	1.19 (3H, s)	16.5 (q)	1.08 (3H, s)
13	112.8 (t)	4.97 (1H, s)	16.4 (q)	1.20 (3H, s)
		4.85 (1H, s)		
14	21.6 (q)	1.00 (3H, s)	25.2 (q)	1.31 (3H, s)
15	29.9 (q)	0.98 (3H, s)	30.7 (q)	1.31 (3H, s)

**Table 4:** Antibiofilm activity of compounds **1**–**4** against *S. mutans.*

Sample	MBIC (µg/mL)
	*Streptococcus mutans* ATCC 25175
*n*-Hexane extract *D. acutangulum*	2500
*n*-Hexane extract *D. cauliflorum*	2500
**1**	500
**2**	250
**3**	NI
**4**	250
Chlorhexidine	62.5

NI: not active.

**Table 5:** Molecular docking results targeted enzymes that contributed to biofilm production.

Receptors	Ligands	Binding Affinity (kcal/mol)	Polar Interaction (Amino Acid Residue)	Non-Polar Interaction
Sortase A/SrtA (PDB ID: 4TQX)	Chlorhexidine	−7.931	Phe237 ^a^, Gln239 ^a^, Tyr241 ^ac^, Gln243 ^a^, Phe246 ^ac^	Pi-alkyl/alkyl
	**1**	−6.072	-	Pi-alkyl/alkyl
	**2a**	−6.266	Tyr241 ^a^, Thr167 ^b^	Pi-alkyl/alkyl
	**2b**	−6.072	Arg238 ^b^	Pi-alkyl/alkyl
	**3**	−6.620	-	Pi-alkyl/alkyl, Pi-sigma
	**4**	−6.394	-	Pi-alkyl/alkyl
Glucosyltransferase B/GtfB (PDB ID: 8FK4)	Chlorhexidine	−8.309	Glu489 ^ac^, Asp562 ^ac^, Asp567 ^ac^, Tyr584 ^ac^, Tyr404 ^b^	Pi-alkyl, Pi-Pi stacked
	**1**	−7.166	Glu489 ^a^	Pi-alkyl/alkyl
	**2a**	−7.001	Asn888 ^a^, Gln566 ^b^	-
	**2b**	−6.949	Trp491 ^a^	-
	**3**	−6.674	-	Pi-alkyl/alkyl, Pi-sigma
	**4**	−6.838	-	Pi-alkyl/alkyl

a: Conventional hydrogen bond, b: carbon–hydrogen bond, c: attractive charge/Pi-cation.

## Data Availability

The original contributions presented in this study are included in the article/Supplementary Material. Further inquiries can be directed to the corresponding author.

## Supplementary Materials

The following supporting information can be downloaded at: https://www.mdpi.com/article/10.3390/molecules31111893/s1, Figure S1: HRTOFMS spectrum of **1**; Figure S2: FTIR spectrum of **1**; Figure S3: ^1^H-NMR spectrum of **1**; Figure S4: ^13^C-NMR and DEPT-135° spectrum of **1**; Figure S5: HRTOFMS spectrum of **2**; Figure S6: ^1^H-NMR spectrum of **2**; Figure S7: ^13^C-NMR and DEPT-135° spectrum of **2**; Figure S8: HSQC spectrum of **2**; Figure S9: ^1^H−^1^H-COSY spectrum of **2**; Figure S10: HMBC spectrum of **2**; Figure S11: NOESY spectrum of **2**. Figure S12: HRTOFMS spectrum of **3**; Figure S13: FTIR spectrum of **3**; Figure S14: ^1^H-NMR spectrum of **3**; Figure S15: ^13^C-NMR and DEPT-135° spectrum of **3**; Figure S16: HRTOFMS spectrum of **4**; Figure S17: FTIR spectrum of **4**; Figure S18: ^1^H-NMR spectrum of **4**; Figure S19: ^13^C-NMR and DEPT-135° spectrum of **4**.

## References

[1] Celik K., Togar B., Turkez H., Taspinar N. (2014). In vitro cytotoxic, genotoxic, and oxidative effects of acyclic sesquiterpene farnesene. Turk. J. Biol..

[2] Buckle J. (2015). Clinical Aromatherapy: Essential Oil in Practice.

[3] Li X., Zhang M., Liu Y., Feng Y., Yang J., Xie Y., Li Y., Xu Y., Huang Z. (2026). Recent advances in the development of bergamot (*Citrus bergamia* Risso) essential oil: From extraction, chemical composition, function to potential applications. Ind. Crops Prod..

[4] Zheng Q., Zhu H., Yang H.P., Peng C., Wu G.X., Zhou Q.M., Xiong L. (2025). Guaiane-type sesquiterpenoids from patchouli oil and their potential anti-depressive effects. Phytochemistry.

[5] Lin S.H., Diao N., Wang Y., Liang D. (2025). Structurally diverse sesquiterpenoid glycosides with anti-inflammatory activity from *Cissampelopsis spelaeicola*. Phytochemistry.

[6] Li B., Zhou S., Wu W., Tian Y., Ren Y., Ma J., Zang Y., Yuan Y., Zhang D., Li C. (2025). Atractylodimers A–D, unprecedented sesquiterpenoid dimers with cage-like skeletons from *Atractylodes macrocephala* and their neuroprotective activities. Chin. J. Nat. Med..

[7] Zhang F.Y., Gan L., Hu X.Y., Zhu G.H., Liao Y.L., Huang G.S., Guo D., Li W. (2026). Saglabranoids A–J, structurally diverse sesquiterpenoids with anti-hepatic fibrosis activity from the roots of *Sarcandra glabra*. J. Mol. Struct..

[8] Wang M., Zhao L., Chen K., Shang Y., Wu J., Guo X., Chen Y., Liu H., Tan H., Qiu S.X. (2020). Antibacterial sesquiterpenes from the stems and roots of *Thuja sutchuenensis*. Bioorganic Chem..

[9] Wang Z.X., Kong W.Z., Guan S.N., Zhang N., Yu Y.B., Zhang X.Y. (2024). Pitsubcosides M–S: Novel antibacterial cadinane sesquiterpenoid glycoside esters from *Pittosporum subulisepalum*. Ind. Crops Prod..

[10] Rahim F.A.M., Salleh W.M.N.H.W., Arzmi M.H., Salihu A.S. (2024). Chemical composition, antifungal, antibiofilm, and molecular docking studies of *Syzygium dyerianum* essential oil. Z. Naturforschung C.

[11] da Cruz R.P., Almeida-Bezerra J.W., Alves D.S., da Silva A.R.P., de Oliveira M.G., Alencar G.G., Coutinho H.D.M., Morais-Braga M.F.B., da Silva M.V. (2026). Chemical composition, antibiofilm activity, and antibacterial potential in vitro and in a zebrafish model of *Myrciaria pilosa* Sobral & Couto essential oil. J. Ethnopharmacol..

[12] Azeem K., Fatima S., Ali A., Ubaid A., Husain F.M., Abid M. (2025). Biochemistry of bacterial biofilm: Insights into antibiotic resistance mechanisms and therapeutic intervention. Life.

[13] Perry E.K., Tan M.W. (2023). Bacterial biofilms in the human body: Prevalence and impacts on health and disease. Front. Cell. Infect. Microbiol..

[14] Khushbu Y., Satyam P. (2016). Dental caries: A review. Asian J. Biomed. Pharm. Sci..

[15] Matsumoto-Nakano M. (2018). Role of *Streptococcus mutans* surface proteins for biofilm formation. Jpn. Dent. Sci. Rev..

[16] Riyadi S.A., Naini A.A., Supratman U. (2023). Sesquiterpenoids from Meliaceae family and their biological activities. Molecules.

[17] Xia M.-J., Zhang M., Li S.-W., Cai Z.-F., Zhao T.-S., Liu A.-H., Luo J., Zhang H.-Y., Li J., Guo Y.-W. (2022). Anti-inflammatory and PTP1B inhibitory sesquiterpenoids from the twigs and leaves of *Aglaia lawii*. Fitoterapia.

[18] Naini A.A., Mayanti T., Supratman U. (2022). Triterpenoids from *Dysoxylum* genus and their biological activities. Arch. Pharm. Res..

[19] Parulian S.S., Nurlelasari N., Naini A.A., Hilmayanti E., Mayanti T., Harneti D., Darwati D., Maharani R., Farabi K., Supratman U. (2022). Sesquiterpenoids from stem bark of *Chisocheton lasiocarpus* and their cytotoxic activity against MCF-7 breast cancer cell. Molekul.

[20] Kouame C., Ouattara Z.A., Kambire D.A., Monteil M., Mamyrbekova J.A., Bighelli A., Tomi F., Lecouvey M., Bekro Y.A. (2022). Chemical composition and biological activity of *Guarea cedrata* leaf and root bark essential oil. Int. J. Biochem. Res. Rev..

[21] Adeniyi B.A., Adagbasa O.O., Idowu P.A., Igbokwe C.O., Moody J.O., Aiyelaagbe O.O. (2024). Extracts of *Trichilia heudelotii* (Meliaceae), a Nigerian medicinal plant, have antibacterial and antifungal activity. J. Pharm. Res. Int..

[22] Fadhilah K., Wahyuono S., Astuti P.A. (2021). Sesquiterpene aldehyde isolated from ethyl acetate extract of *Lansium domesticum* fruit peel. Indones. J. Pharm..

[23] Nugroho A.E., Sugiura R., Momota T., Hirasawa Y., Wong C.P., Kaneda T., Hadi A.H.A., Morita H. (2015). Dysosesquiflorins A and B, sesquiterpenoids from *Dysoxylum densiflorum*. J. Nat. Med..

[24] Naini A.A., Mayanti T., Nurlelasari, Harneti D., Maharani R., Safari A., Hidayat A.C., Farabi K., Lesmana R., Supratman U. (2022). Cytotoxic sesquiterpenoids from *Dysoxylum parasiticum* (Osbeck) Kosterm. stem bark. Phytochem. Lett..

[25] Naini A.A., Mayanti T., Harneti D., Darwati D., Nurlelasari N., Maharani R., Farabi K., Herlina T., Supratman U., Fajriah S. (2023). Sesquiterpenoids and sesquiterpenoid dimers from the stem bark of *Dysoxylum parasiticum*. Phytochemistry.

[26] Gu J., Cheng G.G., Qian S.Y., Li Y., Liu Y.P., Luo X.D. (2014). Dysoxydensins A–G, seven new clerodane diterpenoids from *Dysoxylum densiflorum*. Planta Med..

[27] Zhang P.Z., Zhang Y.M., Lin Y., Wang F., Zhang G.L. (2020). Three new diterpenes from *Dysoxylum lukii* and their NO production inhibitory activity. J. Asian Nat. Prod. Res..

[28] Ragasa C.Y., Torres O.B., Bernardo L.O., Mandia E.H., Don M.J., Shen C.C. (2013). Glabretal-type triterpenoids from *Dysoxylum mollissimum*. Phytochem. Lett..

[29] He X.F., Wang X.N., Yin S., Dong L., Yue J.M. (2011). Ring A-seco triterpenoids with antibacterial activity from *Dysoxylum hainanense*. Bioorganic Med. Chem. Lett..

[30] Bhardwaj N., Gupta P., Tripathi N., Chakrabarty S., Verma A., Kumari S., Gautam V., Ravikanth G., Jain S.K. (2024). New ring-A modified cycloartane triterpenoids from *Dysoxylum malabaricum* bark: Isolation, structure elucidation and cytotoxicity. Steroids.

[31] Yan H.J., Wang J.S., Kong L.Y. (2014). Cytotoxic dammarane-type triterpenoids from the stem bark of *Dysoxylum binecteriferum*. J. Nat. Prod..

[32] Xu J., Ni G., Yang S., Yue J. (2013). Dysoxylumasins A–F: Six new limonoids from *Dysoxylum mollissimum* Bl. *Chin*. J. Chem..

[33] Liu W.X., Tang G.H., He H.P., Zhang Y., Li S.L., Hao X.J. (2012). Limonoids and triterpenoids from the twigs and leaves of *Dysoxylum hainanense*. Nat. Prod. Bioprospect..

[34] Naini A.A., Mayanti T., Maharani R., Harneti D., Nurlelasari N., Farabi K., Fajriah S., Hilmayanti E., Kabayama K., Shimoyama A. (2024). Paraxylines A–G: Highly oxygenated preurianin-type limonoids with immunomodulatory TLR4 and cytotoxic activities from the stem bark of *Dysoxylum parasiticum*. Phytochemistry.

[35] Riyadi S.A., Naini A.A., Mayanti T., Farabi K., Harneti D., Nurlelasari N., Maharani R., Lesmana R., Fajriah S., Jungsuttiwong S. (2024). Alliaceumolide A: A rare undescribed 17-membered macrolide from Indonesian *Dysoxylum alliaceum*. Phytochem. Lett..

[36] Nishizawa M., Inoue A., Sastrapradja S., Hayashi Y. (1983). (+)-8-Hydroxycalamenene: A fish-poison principle of *Dysoxylum acutangulum* and D. Alliaceum. Phytochemistry.

[37] Ismail I.S., Nagakura Y., Hirasawa Y., Hosoya T., Lazim M.I.M., Lajis N.H., Shiro M., Morita H. (2009). Chrotacumines A–D, chromone alkaloids from *Dysoxylum acutangulum*. J. Nat. Prod..

[38] Lazim M.I.M., Ismail I.S., Shaari K., Abd. Latip J., Al-Mekhlafi N.A., Morita H. (2013). Chrotacumines E and F, two new chromone-alkaloid analogs from *Dysoxylum acutangulum* (Meliaceae) leaves. Chem. Biodivers..

[39] Morita H., Nugroho A.E., Nagakura Y., Hirasawa Y., Yoshida H., Kaneda T., Shirota O., Ismail I.S. (2014). Chrotacumines G–J, chromone alkaloids from *Dysoxylum acutangulum* with osteoclast differentiation inhibitory activity. Bioorg. Med. Chem. Lett..

[40] Ismail I.S., Nagakura Y., Hirasawa Y., Hosoya T., Lazim M.I.M., Lajis N.H., Morita H. (2009). Acutaxylines A and B, two novel triterpenes from *Dysoxylum acutangulum*. Tetrahedron Lett..

[41] Salleh W.M.N.H.W., Khamis S., Tawang A. (2021). Chemical composition of the essential oil of *Dysoxylum cauliflorum* Hiern (Meliaceae). Nat. Volatiles Essent. Oils.

[42] Huang R., Harrison L.J., Sim K.Y. (1999). A triterpenoid with a novel abeo-dammarane skeleton from *Dysoxylum cauliflorum*. Tetrahedron Lett..

[43] Dharmayani N.K.T., Yoshimura T., Hermawati E., Juliawaty L.D., Syah Y.M. (2020). Antibacterial and antifungal two phenolic sesquiterpenes from *Dysoxylum densiflorum*. Z. Naturforschung C.

[44] Feliciano A.S., Medarde M., Gordaliza M., Del Olmo E., Miguel del Corral J.M. (1989). Sesquiterpenoids and phenolics of *Pulicaria paludosa*. Phytochemistry.

[45] Gunawan L., Mustofa H.N., Naini A.A., Harneti D., Hidayat A.T., Nurlelasari N., Maharani R., Mayanti T., Fajriah S., Awang K. (2025). Sesquiterpenoids from *Dysoxylum amooroides* stem bark: Isolation, structure determination, and cytotoxicity against MCF-7 breast cancer cells. Indones. J. Chem..

[46] Aguilar-Guadarrama A.B., Rios M.Y. (2004). Three new sesquiterpenes from *Croton arboreous*. J. Nat. Prod..

[47] Jares E.A., Potolovskii L.A., Katrenko T.I., Polyakova A.A., Fufaev A.A., Bessonova R.N., Terweij-Groen C.P., Heemstra S., Kraak J.C. (1989). Isolation of sesquiterpenes from *Senecio crassiflorus* by combined dry column and high performance liquid chromatography. J. High Resolut. Chromatogr..

[48] Misra L.N., Jakupovic J., Bohlmann F., Schmeda-Hirschmann G. (1985). Isodaucane derivatives, norsesquiterpenes and clerodanes from *Chromolaena laevigata*. Tetrahedron.

[49] Bülow N., König W.A. (2000). The role of germacrene D as a precursor in sesquiterpene biosynthesis: Investigations of acid-catalyzed, photochemical and thermally induced rearrangements. Phytochemistry.

[50] Qin G.F., Tang X.L., Sun Y.T., Luo X.C., Zhang J., Ofwegen L.V., Sung P.J., Li P.L., Li G.Q. (2018). Terpenoids from the soft coral *Sinularia* sp. collected in Yongxing Island. Mar. Drugs.

[51] Kautsari A., Naini A.A., Riyadi S.A., Mayanti T., Harizon H., Fajriah S., Supratman U. (2024). The sesquiterpenoids from the stem bark of *Dysoxylum excelsum* and their cytotoxic activities against HeLa cancer cell lines. Molekul.

[52] Heymann H., Tezuka Y., Kikuchi T., Supriyatna S. (1994). Constituents of *Sindora sumatrana* Miq. I. Isolation and NMR spectral analysis of sesquiterpenes from the dried pods. Chem. Pharm. Bull..

[53] Kitaoka N., Lu X., Yang B., Peters R.J. (2015). The application of synthetic biology to elucidation of plant mono-, sesqui-, and diterpenoid metabolism. Mol. Plant.

[54] Welsch M.E., Kaplan A., Chambers J.M., Stokes M.E., Bos P.H., Zask A., Zhang Y., Sanchez-Martin M., Badgley M.A., Huang C.S. (2017). Multivalent small-molecule pan-RAS inhibitors. Cell.

[55] Gonçalves O., Pereira R., Gonçalves F., Mendo S., Coimbra M.A., Rocha S.M. (2011). Evaluation of the mutagenicity of sesquiterpenic compounds and their influence on the susceptibility towards antibiotics of two clinically relevant bacterial strains. Mutat. Res. Genet. Toxicol. Environ..

[56] Simões M., Rocha S., Coimbra M.A., Vieira M.J. (2008). Enhancement of *Escherichia coli* and *Staphylococcus aureus* antibiotic susceptibility using sesquiterpenoids. Med. Chem..

[57] Curvelo J.A.R., Marques A.M., Barreto A.L.S., Romanos M.T.V., Portela M.B., Kaplan M.A.C., Soares R.M.A. (2014). A novel nerolidol-rich essential oil from *Piper claussenianum* modulates *Candida albicans* biofilm. J. Med. Microbiol..

[58] Cho E., Hwang J.Y., Park J.S., Oh D., Oh D.C., Park H.G., Shin J., Oh K.B. (2022). Inhibition of *Streptococcus mutans* adhesion and biofilm formation with small-molecule inhibitors of sortase A from *Juniperus chinensis*. J. Oral Microbiol..

[59] Nijampatnam B., Ahirwar P., Pukkanasut P., Womack H., Casals L., Zhang H., Cai X., Michalek S.M., Wu H., Velu S.E. (2020). Discovery of potent inhibitors of *Streptococcus mutans* biofilm with antivirulence activity. ACS Med. Chem. Lett..

[60] Ren Z., Cui T., Zeng J., Chen L., Zhang W., Xu X., Cheng L., Li M., Li J., Zhou X. (2016). Molecule targeting glucosyltransferase inhibits *Streptococcus mutans* biofilm formation and virulence. Antimicrob. Agents Chemother..

