# Supplementary material for: Sesquiterpenoids from Dysoxylum acutangulum Miq. and Dysoxylum cauliflorum Hiern Twigs: Antibiofilm Activity Against Streptococcus mutans
Source: Molecules. 2026 Jun 1;31(11):1893. doi: 10.3390/molecules31111893 (PMC13257915; doi:10.3390/molecules31111893)
Supplement: Supplementary file 1 [file molecules-31-01893-s001.zip › molecules-4302434-supplementary.pdf]

Supplementary Materials

Sesquiterpenoids from *Dysoxylum acutangulum* Miq. and *Dysoxylum cauliflorum* Hiern Twigs: Antibiofilm Activity Against *Streptococcus mutans*

Risyandi Anwar [1,*], Hikma Ainazzahra [2,3], Citra Nisa Ul Inayah [2], Al Arofatus Naini [3], Endang Juliansyah [2], Elpri Eka Permadi [3], Aditya Nugroho [4], Kindi Farabi [2] and Unang Supratman [2,5]

[1] Herbal Medicine Research, Department of Pediatric Dentistry, Faculty of Dental Medicine, University of Muhammadiyah Semarang, Semarang 50272, Indonesia
[2] Department of Chemistry, Faculty of Mathematics and Natural Sciences, Universitas Padjadjaran, Jl. Raya Bandung–Sumedang Km. 21, Jatinangor, Sumedang 45363, Indonesia; hikma21001@mail.unpad.ac.id (H.A.); citra21003@mail.unpad.ac.id (C.N.U.I.); endang18001@mail.unpad.ac.id (E.J.); kindi.farabi@unpad.ac.id (K.F.); unang.supratman@unpad.ac.id (U.S.)
[3] Research Center for Pharmaceutical Ingredients and Traditional Medicine, National Research and Innovation Agency (BRIN), Cibinong Science Center Complex-BRIN, Cibinong, Bogor 16911, Indonesia; alar002@brin.go.id (A.A.N.); elpri.eka.permadi@brin.go.id (E.E.P.)
[4] Research Center for Applied Botany, National Research and Innovation Agency (BRIN), Cibinong Science Center Complex-BRIN, Cibinong, Bogor 16911, Indonesia; adit035@brin.go.id
[5] Central Laboratory, Universitas Padjadjaran, Jl. Raya Bandung–Sumedang Km. 21, Jatinangor, Sumedang 45363, Indonesia
* Correspondence: drg.risyandi@unimus.ac.id; Tel.: +62-24-76740293

Contents

Figure S1. HRTOFMS Spectrum of **1**.
Figure S2. FTIR Spectrum of **1**.
Figure S3. ^{1}H-NMR Spectrum of **1** (700 MHz in $CDCl_3$).
Figure S4. ^{13}C-NMR and DEPT-135° Spectrum of **1** (175 MHz in $CDCl_3$).
Figure S5. HRTOFMS Spectrum of **2**.
Figure S6. ^{1}H-NMR Spectrum of **2** (700 MHz in $CDCl_3$).
Figure S7. ^{13}C-NMR and DEPT-135° Spectrum of **2** (175 MHz in $CDCl_3$).
Figure S8. HSQC Spectrum of **2**.
Figure S9. ^{1}H-^{1}H-COSY Spectrum of **2**.
Figure S10. HMBC Spectrum of **2**.
Figure S11. NOESY Spectrum of **2**.
Figure S12. HRTOFMS Spectrum of **3**.
Figure S13. FTIR Spectrum of **3**.
Figure S14. ^{1}H-NMR Spectrum of **3** (700 MHz in $CDCl_3$).
Figure S15. ^{13}C-NMR and DEPT-135° Spectrum of **3** (175 MHz in $CDCl_3$).
Figure S16. HRTOFMS Spectrum of **4**.
Figure S17. FTIR Spectrum of **4**.
Figure S18. ^{1}H-NMR Spectrum of **4** (700 MHz in $CDCl_3$).
Figure S19. ^{13}C-NMR and DEPT-135° Spectrum of **4** (175 MHz in $CDCl_3$).

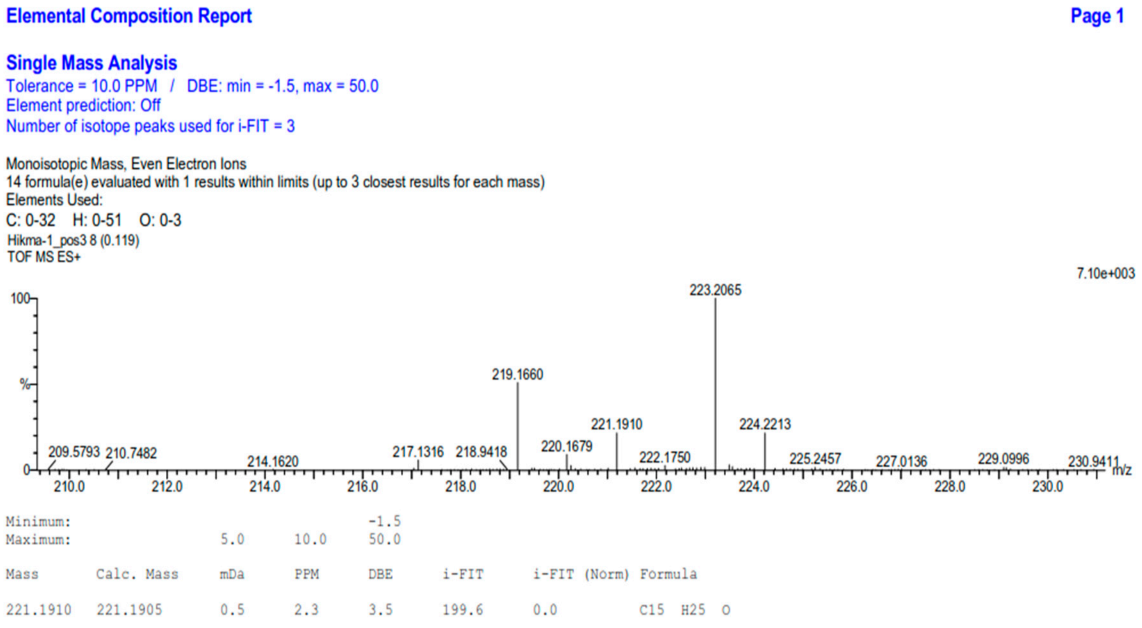

Elemental Composition Report Page 1

Single Mass Analysis
Tolerance = 10.0 PPM / DBE: min = -1.5, max = 50.0
Element prediction: Off
Number of isotope peaks used for i-FIT = 3

Monoisotopic Mass, Even Electron Ions
14 formula(e) evaluated with 1 results within limits (up to 3 closest results for each mass)
Elements Used:
C: 0-32 H: 0-51 O: 0-3
Hikma-1_pos3 8 (0.119)
TOF MS ES+

Minimum:				-1.5			
Maximum:		5.0	10.0	50.0			
Mass	Calc. Mass	mDa	PPM	DBE	i-FIT	i-FIT (Norm)	Formula
221.1910	221.1905	0.5	2.3	3.5	199.6	0.0	C15 H25 O

Figure S1. HRTOFMS Spectrum of **1**.

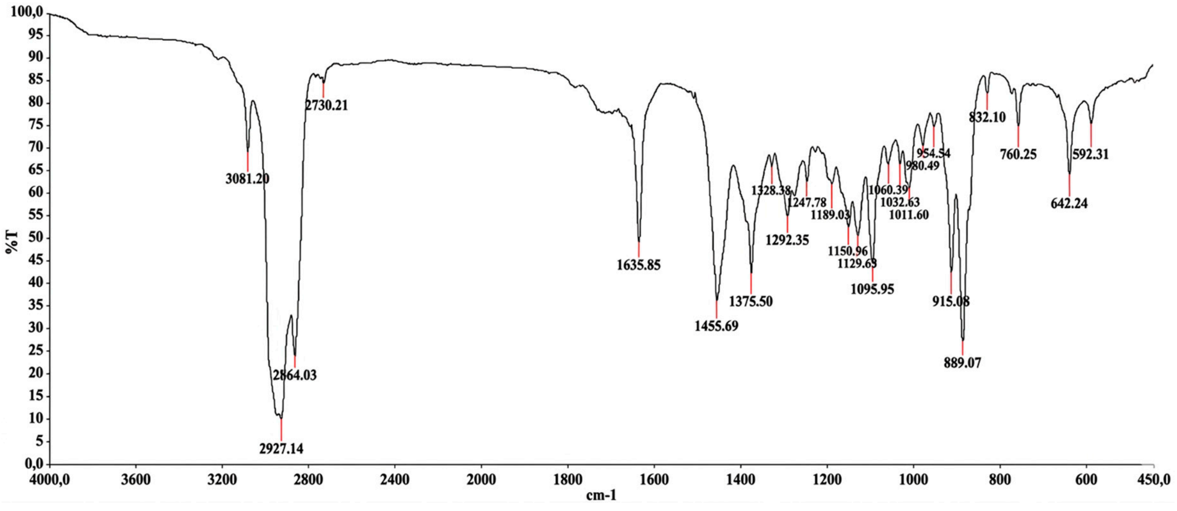

Figure S2. FTIR Spectrum of **1**.

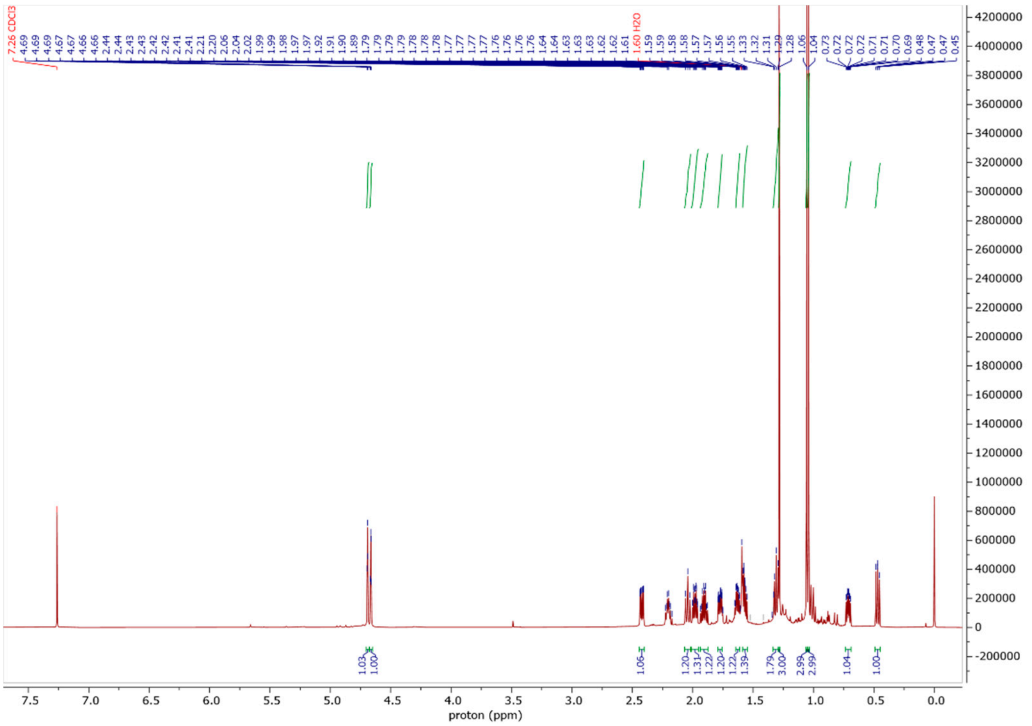

Figure S3. ^{1}H-NMR Spectrum of **1** (700 MHz in $CDCl_3$).

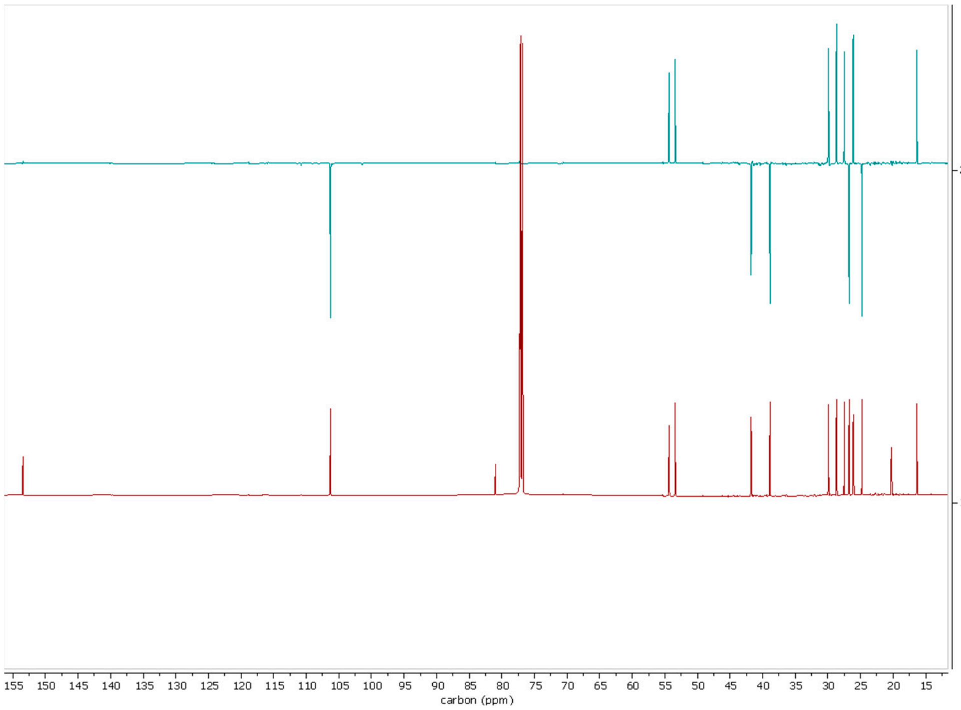

Figure S4. ^{13}C-NMR and DEPT-135° Spectrum of **1** (175 MHz in $CDCl_3$).

Elemental Composition Report Page 1

Single Mass Analysis
Tolerance = 30.0 mDa / DBE: min = -1.5, max = 50.0
Element prediction: Off
Number of isotope peaks used for i-FIT = 3

Monoisotopic Mass, Even Electron Ions
72 formula(e) evaluated with 10 results within limits (up to 50 closest results for each mass)
Elements Used:
C: 0-500 H: 0-1000 O: 0-200 K: 0-1
TAN 4 2 (0.051) Cm (2)
TOF MS ES+

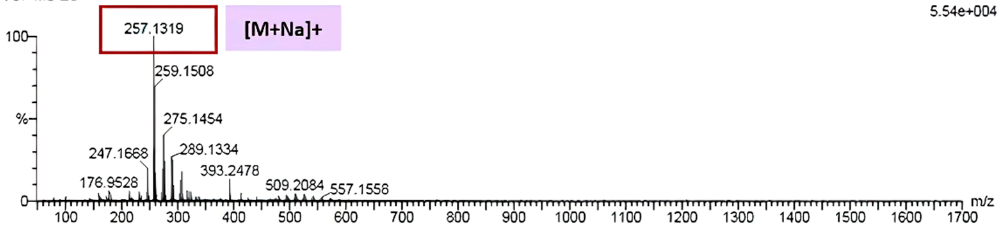

Minimum:				-1.5			
Maximum:		30.0	10.0	50.0			
Mass	Calc. Mass	mDa	PPM	DBE	i-FIT	i-FIT (Norm)	Formula
257.1319	257.1330	-1.1	-4.3	12.5	166.4	2.3	C20 H17
	257.1308	1.1	4.3	4.5	166.2	2.0	C15 H22 O2 Na

Figure S5. HRTOFMS Spectrum of **2**.

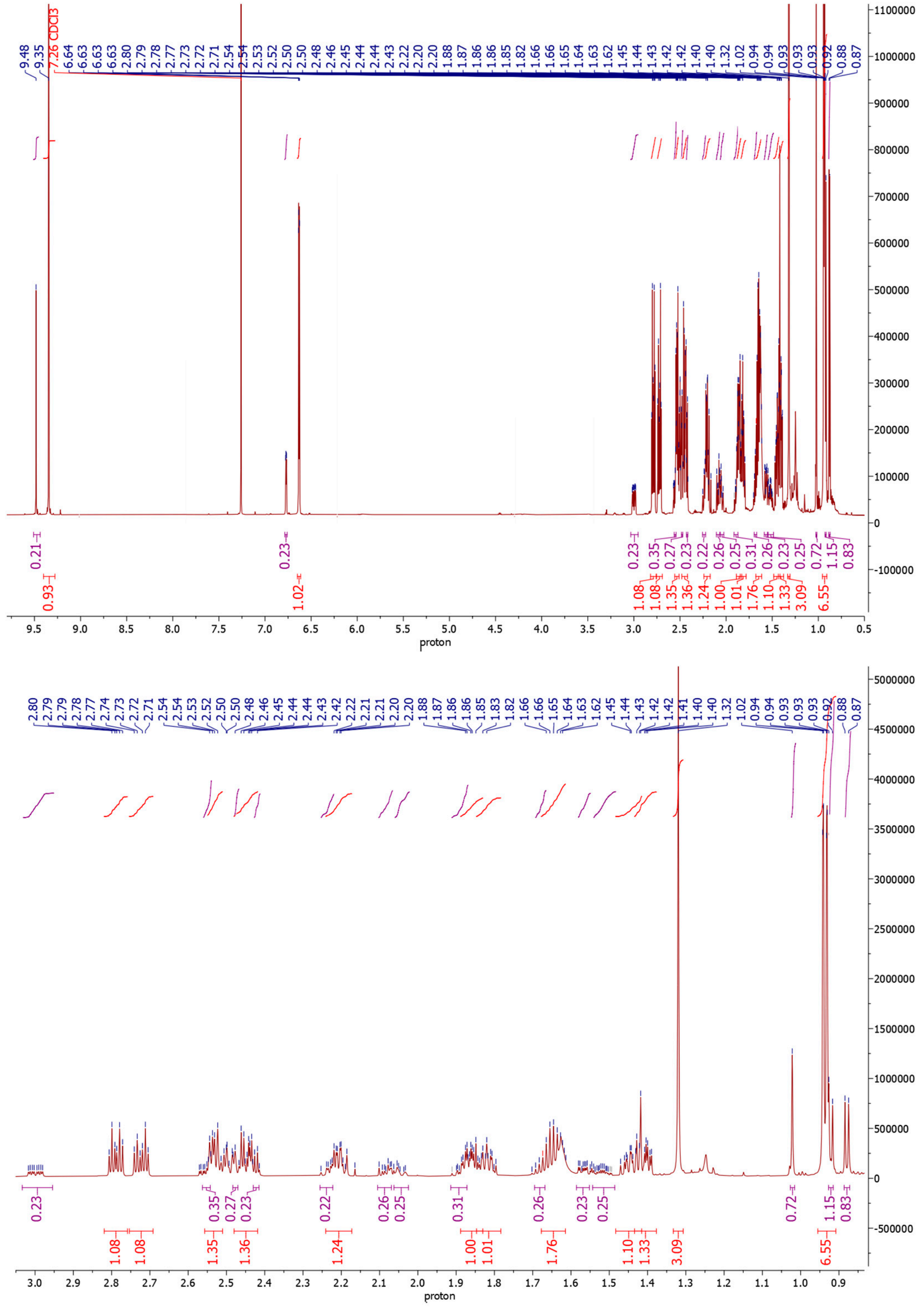

Figure S6. ^{1}H-NMR Spectrum of the mixture **2a** (red) and **2b** (purple) (700 MHz in $CDCl_3$).

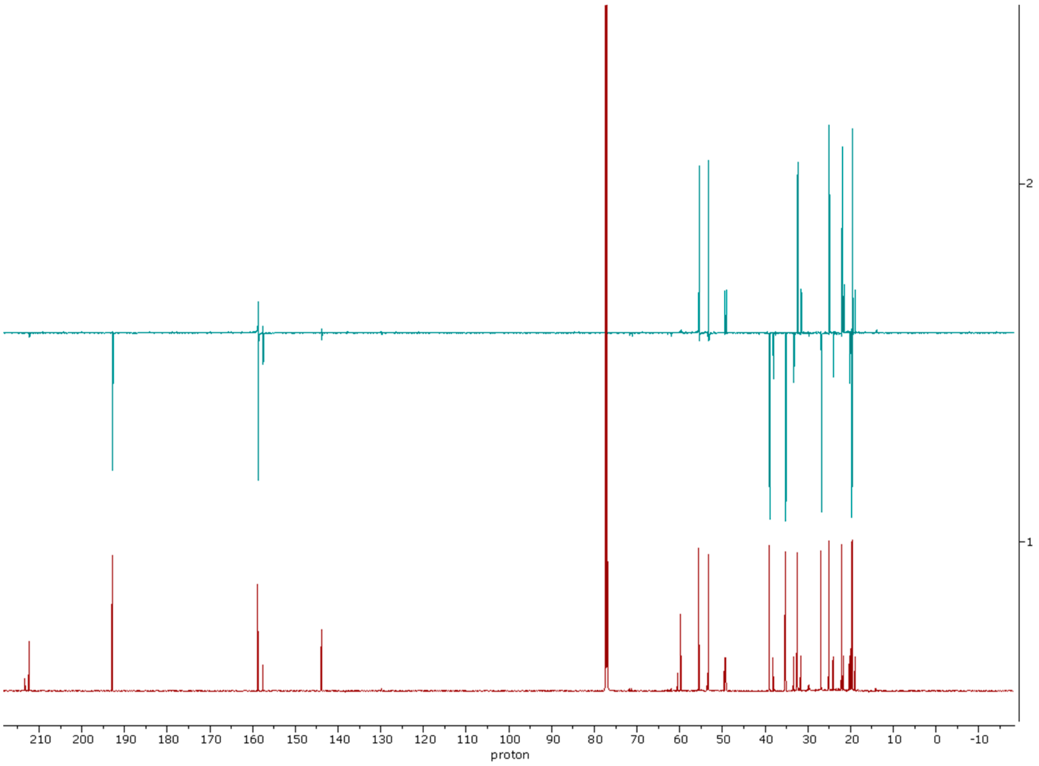

Figure S7. ^{13}C-NMR and DEPT-135° Spectrum of **2** (175 MHz in $CDCl_3$).

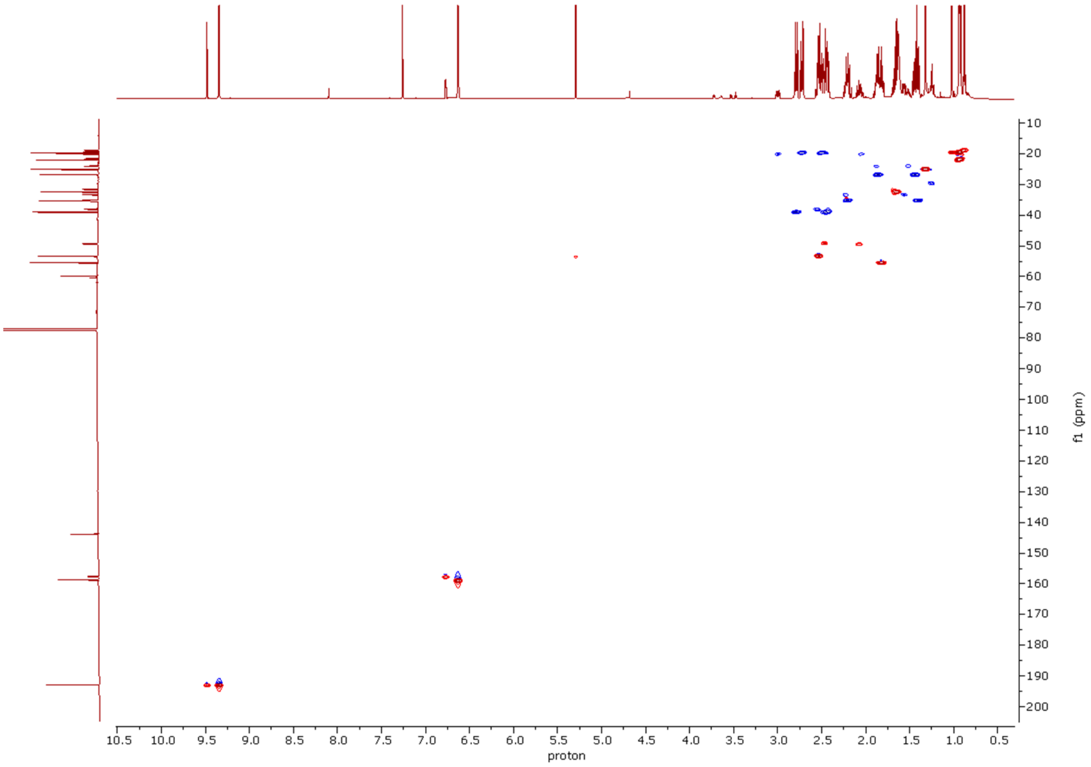

Figure S8. HSQC Spectrum of **2**.

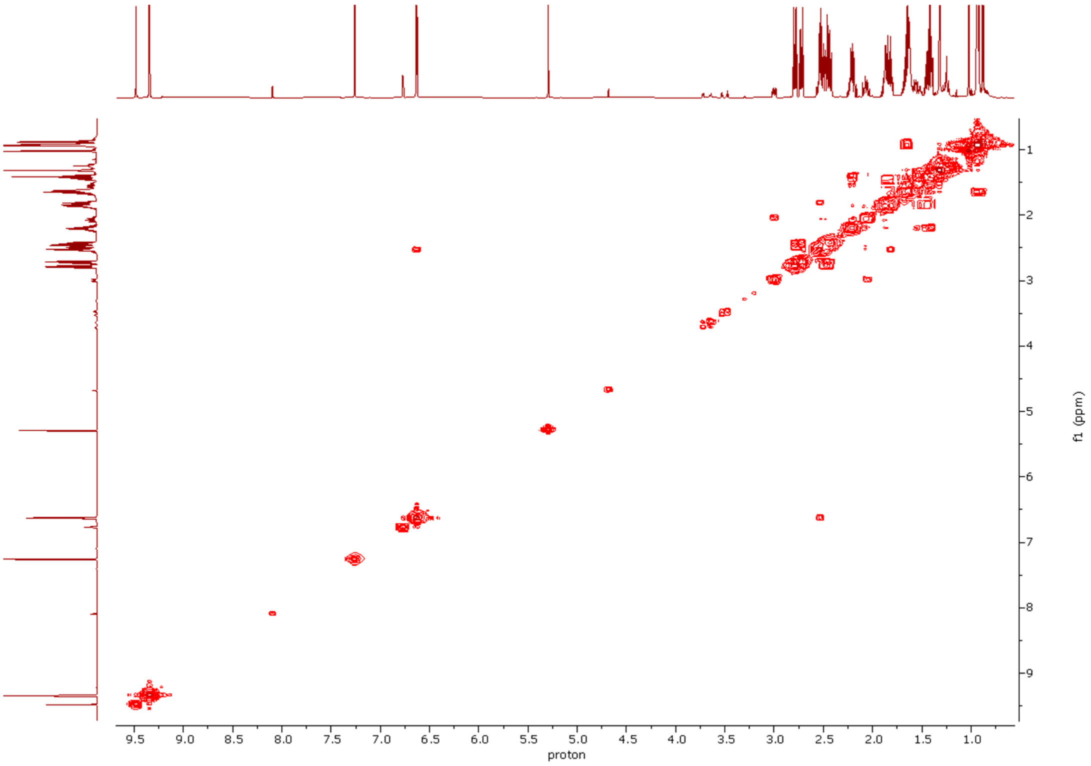

Figure S9. ^1H-^1H-COSY Spectrum of **2**.

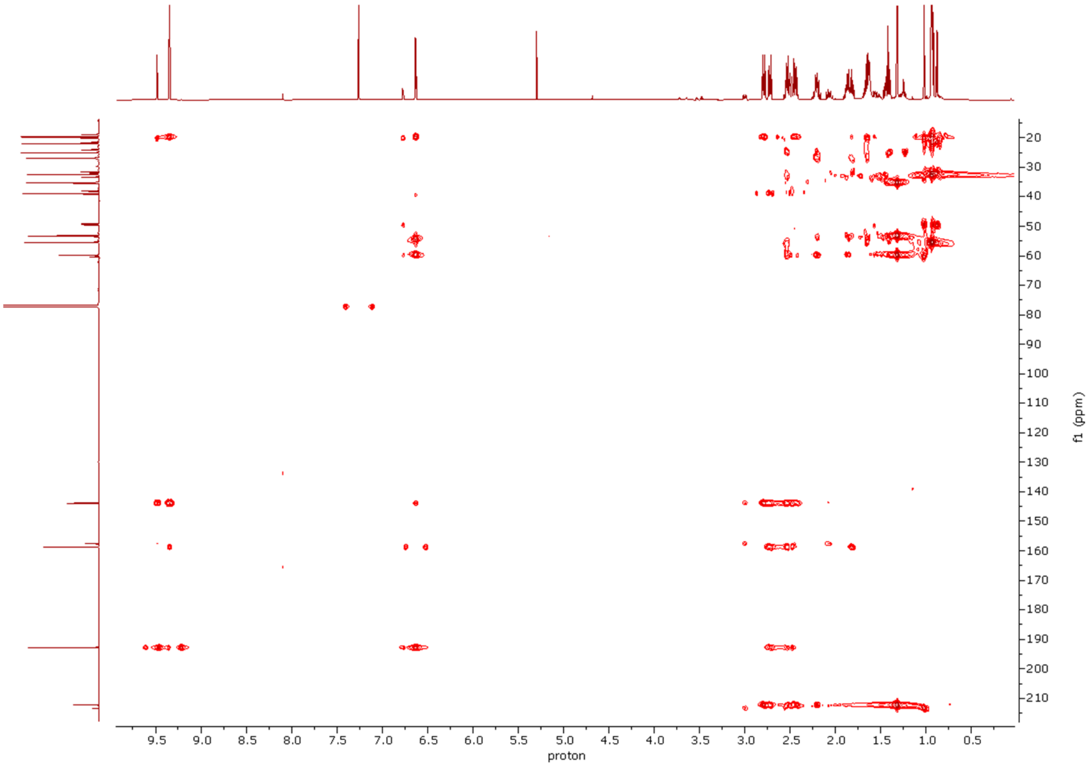

Figure S10. HMBC Spectrum of **2**.

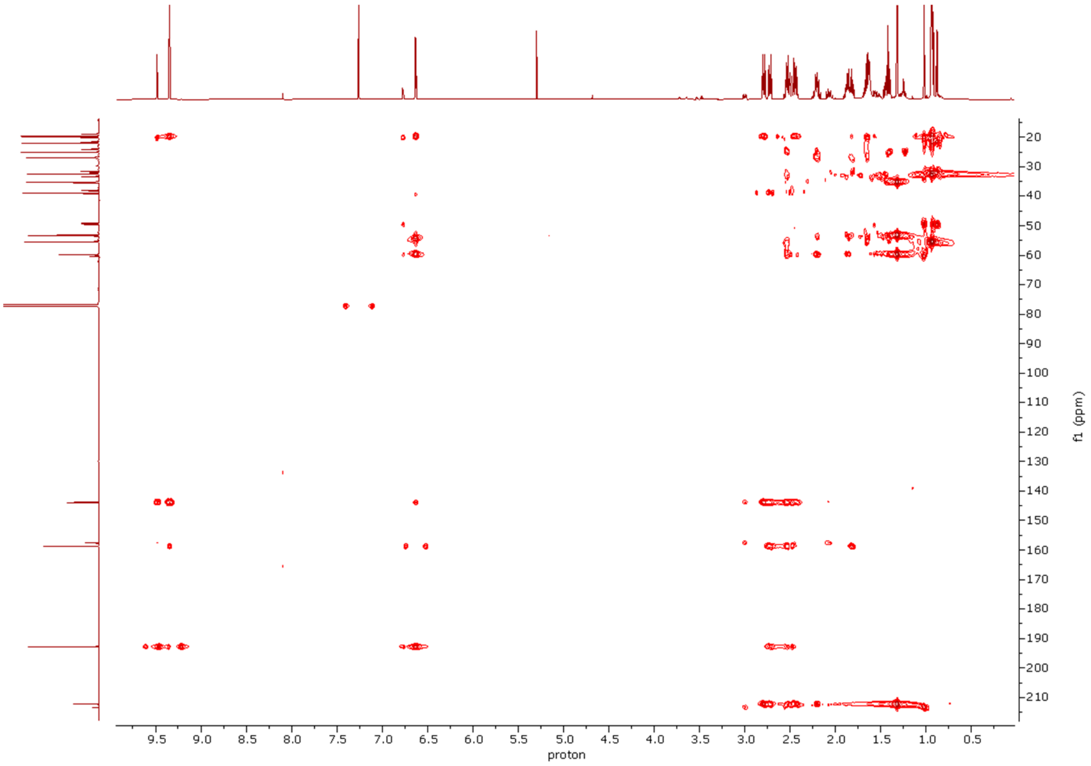

Figure S11. NOESY Spectrum of **2**.

Elemental Composition Report **Page 1**

Single Mass Analysis
Tolerance = 10.0 PPM / DBE: min = -1.5, max = 50.0
Element prediction: Off
Number of isotope peaks used for i-FIT = 3

Monoisotopic Mass, Even Electron Ions
19 formula(e) evaluated with 1 results within limits (up to 3 closest results for each mass)
Elements Used:
C: 0-32 H: 0-51 O: 0-5
Hikma-2_pos 84 (1.411) Cm (84:93)
TOF MS ES+

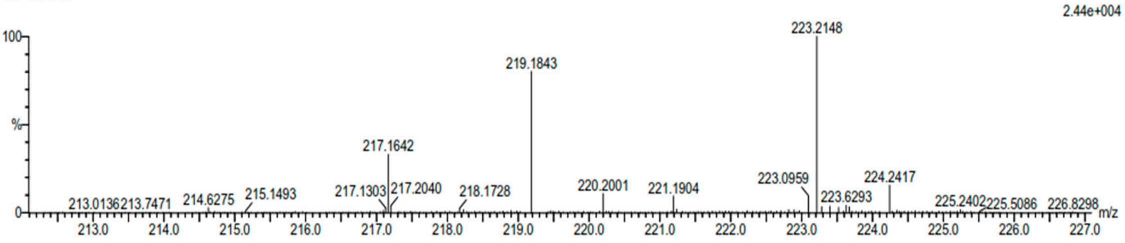

Minimum:				-1.5			
Maximum:		5.0	10.0	50.0			
Mass	Calc. Mass	mDa	PPM	DBE	i-FIT	i-FIT (Norm)	Formula
221.1904	221.1905	-0.1	-0.5	3.5	476.1	0.0	C15 H25 O

Figure S12. HRTOFMS Spectrum of **3**.

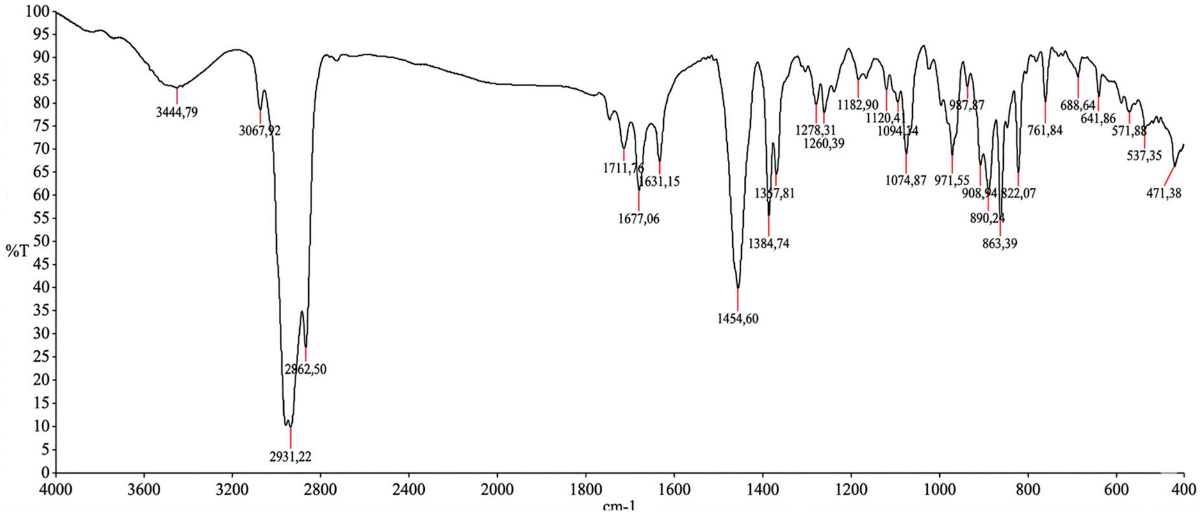

Figure S13. FTIR Spectrum of **3**.

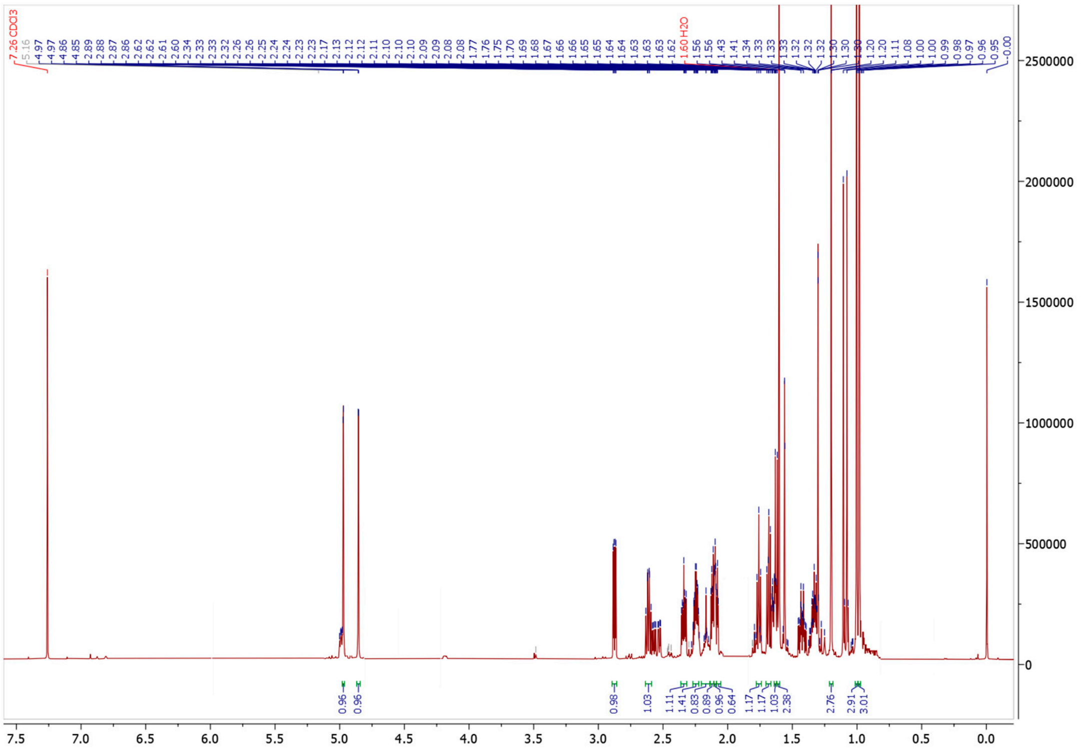

Figure S14. ^{1}H-NMR Spectrum of **3** (700 MHz in $CDCl_3$).

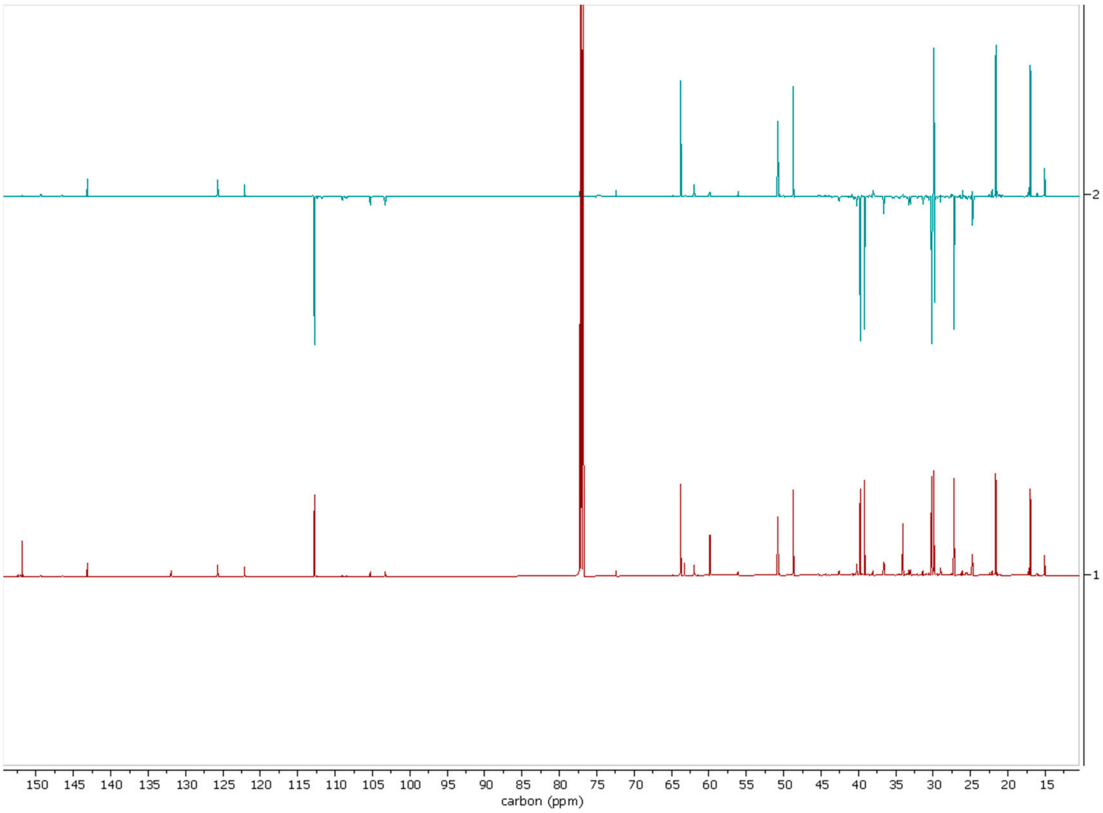

Figure S15. ^{13}C-NMR and DEPT-135° Spectrum of **3** (175 MHz in $CDCl_3$).

Elemental Composition Report **Page 1**

Single Mass Analysis
Tolerance = 10.0 PPM / DBE: min = -1.5, max = 50.0
Element prediction: Off
Number of isotope peaks used for i-FIT = 3

Monoisotopic Mass, Even Electron Ions
14 formula(e) evaluated with 1 results within limits (up to 3 closest results for each mass)
Elements Used:
C: 0-32 H: 0-51 O: 0-3
Citra-2_pos3 6 (0.085)
TOF MS ES+

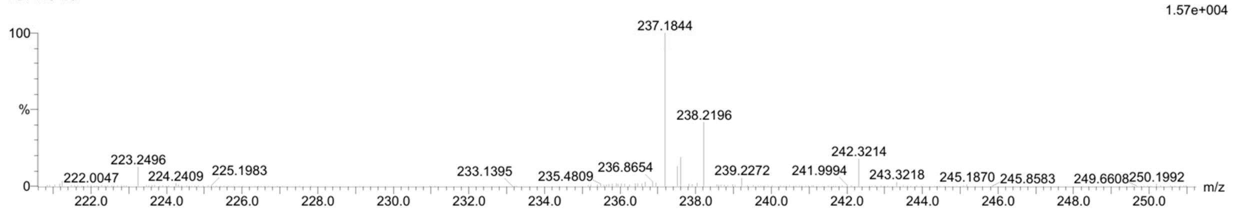

Minimum:				-1.5			
Maximum:		5.0	10.0	50.0			
Mass	Calc. Mass	mDa	PPM	DBE	i-FIT	i-FIT (Norm)	Formula
237.1844	237.1855	-1.1	-4.6	3.5	190.2	0.0	C15 H25 O2

Figure S16. HRTOFMS Spectrum of **4**.

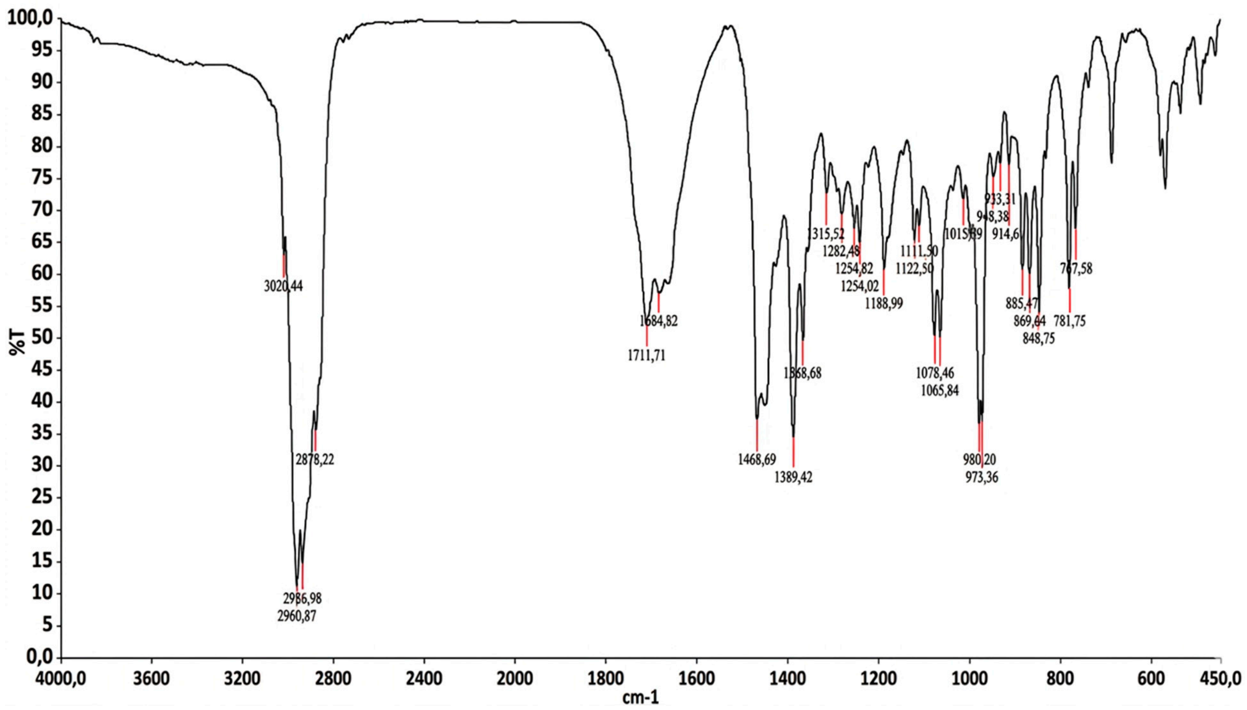

Figure S17. FTIR Spectrum of **4**.

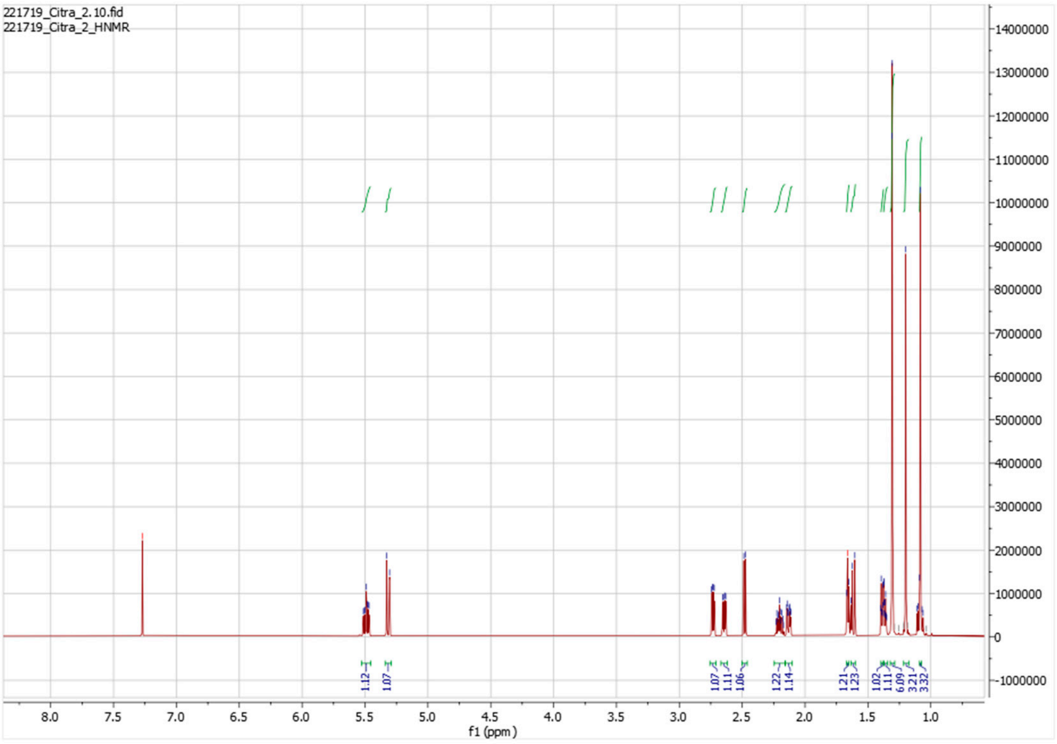

Figure S18. ^{1}H-NMR Spectrum of **4** (700 MHz in $CDCl_3$).

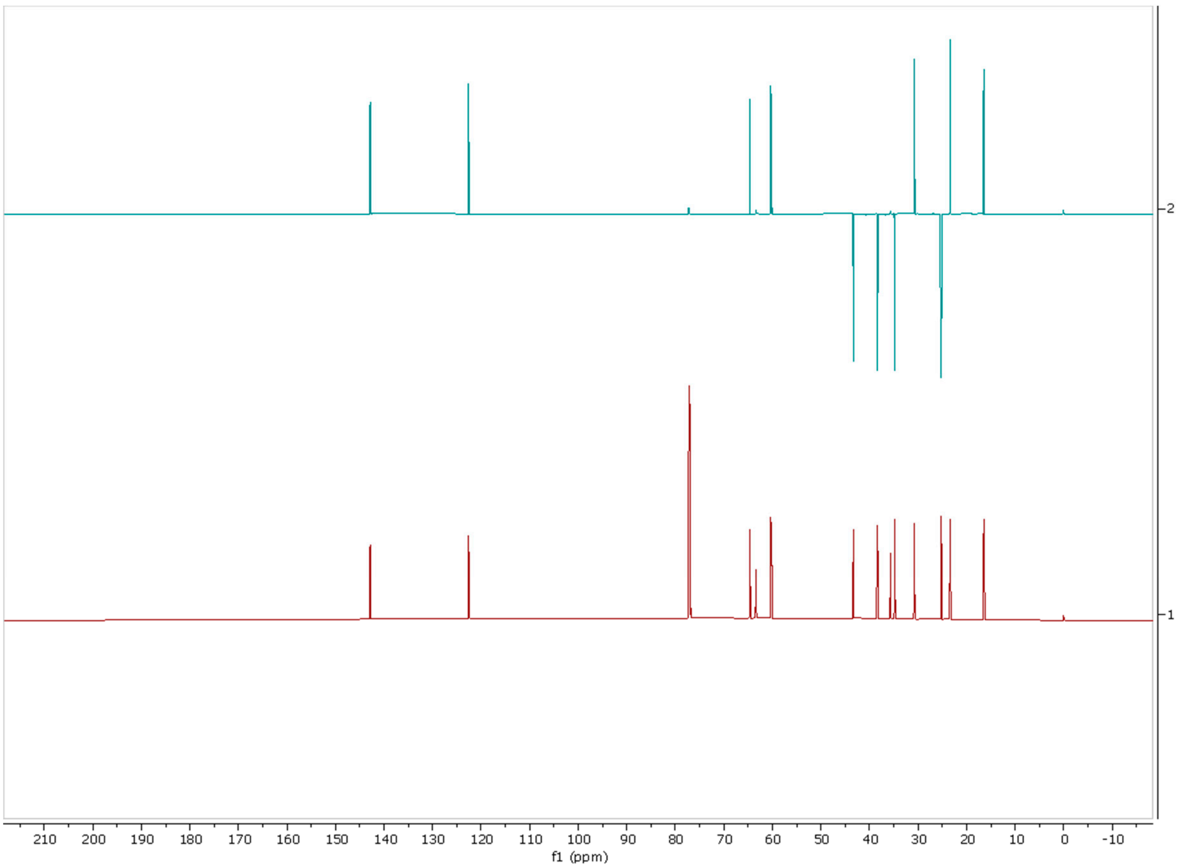

Figure S19. ^{13}C-NMR and DEPT-135° Spectrum of **4** (175 MHz in $CDCl_3$).
